# The Role of Incoherent MicroRNA-Mediated Feedforward Loops in Noise Buffering

**DOI:** 10.1371/journal.pcbi.1001101

**Published:** 2011-03-10

**Authors:** Matteo Osella, Carla Bosia, Davide Corá, Michele Caselle

**Affiliations:** 1 Dipartimento di Fisica Teorica and INFN University of Torino, Torino, Italy; 2 Center for Complex Systems in Molecular Biology and Medicine, University of Torino, Torino, Italy; 3 Systems Biology Lab, Institute for Cancer Research and Treatment (IRCC), School of Medicine, University of Torino, Torino, Italy; ETH Zurich, Switzerland

## Abstract

MicroRNAs are endogenous non-coding RNAs which negatively regulate the expression of protein-coding genes in plants and animals. They are known to play an important role in several biological processes and, together with transcription factors, form a complex and highly interconnected regulatory network. Looking at the structure of this network, it is possible to recognize a few overrepresented motifs which are expected to perform important elementary regulatory functions. Among them, a special role is played by the microRNA-mediated feedforward loop in which a master transcription factor regulates a microRNA and, together with it, a set of target genes. In this paper we show analytically and through simulations that the incoherent version of this motif can couple the fine-tuning of a target protein level with an efficient noise control, thus conferring precision and stability to the overall gene expression program, especially in the presence of fluctuations in upstream regulators. Among the other results, a nontrivial prediction of our model is that the optimal attenuation of fluctuations coincides with a modest repression of the target expression. This feature is coherent with the expected fine-tuning function and in agreement with experimental observations of the actual impact of a wide class of microRNAs on the protein output of their targets. Finally, we describe the impact on noise-buffering efficiency of the cross-talk between microRNA targets that can naturally arise if the microRNA-mediated circuit is not considered as isolated, but embedded in a larger network of regulations.

## Author Summary

The expression of protein-coding genes is controlled by a complex network of regulatory interactions. It is becoming increasingly appreciated that the post-transcriptional repression by microRNAs, a class of small non-coding RNAs, is a key layer of regulation in several biological processes. Since gene expression is a fundamentally stochastic process, the mixed network (comprising transcriptional and microRNA-mediated regulations) has to reliably perform its functions in the presence of noise. In this paper we investigate the function of one of the recurrent architectures of this network, the microRNA-mediated feedforward loops, using a detailed analytical model and simulations. With this approach we show that these regulatory circuits are appropriately designed so as to control noise, giving a rigorous mathematical proof of a previously proposed biological intuition. Moreover the theoretical framework introduced in this paper allows us to make nontrivial predictions that are presently in agreement with observed features of microRNA regulation and that could be more specifically tested experimentally in the future.

## Introduction

MicroRNAs (miRNAs) are endogenous small non-coding RNAs which negatively regulate the protein production of their targets in metazoans and plants. They are expected to target a substantial portion of the human genome [1] and have been shown to play key roles in several biological processes ranging from development and metabolism to apoptosis and signaling pathways [2]–[6]. Moreover their profiles are altered in several human diseases [7], [8], making miRNAs a major focus of research in nowadays molecular biology.

**Figure 1:**
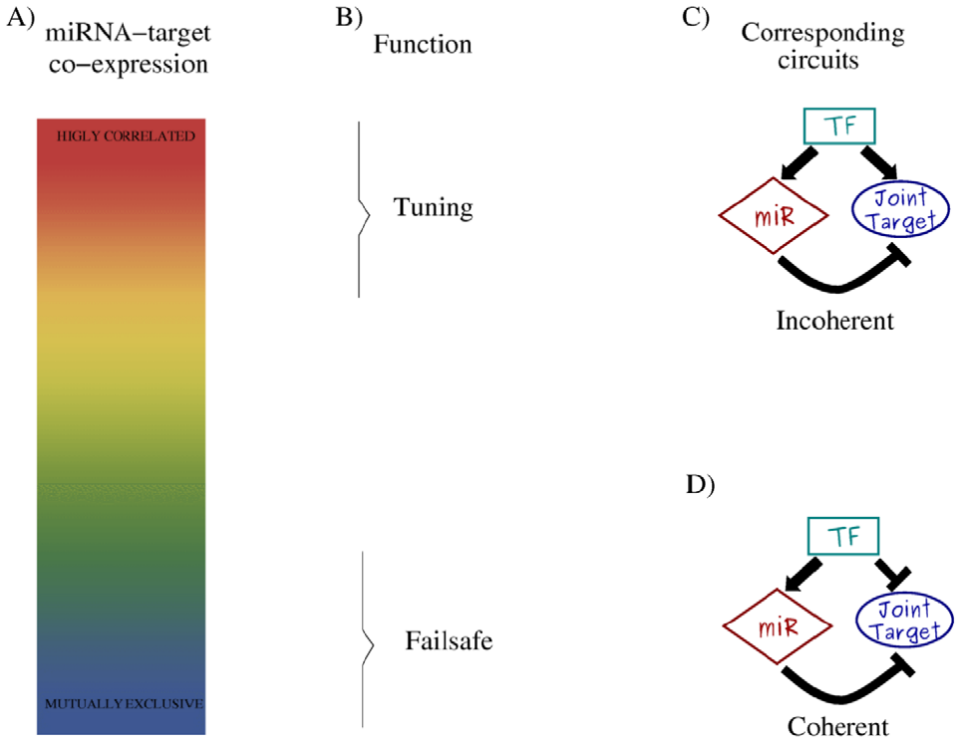
Overview of the connections between miRNA-target expression, miRNA function and regulatory circuitry. (A) MiRNAs and corresponding targets can present different degrees of coexpression between the two extremes of concurrent expression (high correlation) and exclusive domains (high anticorrelation). These two opposite situations are expected to correspond to different functional roles (B) for the miRNA repression. A peculiar expression pattern, evidence of a functional aim, is a consequence of the network structure in which miRNAs are embedded. A high miRNA-target correlation can be achieved through the incoherent FFL (C), where the miRNA repression is opposite to the TF action. Whereas a failsafe control can be performed by a coherent FFL (D), in which the miRNA reinforces the TF action leading to mutually exclusive domains of miRNA-target expression.

Recent work, reviewed in [9], has shown that the actions of miRNAs and transcription factors (TFs) are often highly coordinated, suggesting that the transcriptional and post-transcriptional layers of regulation are strongly correlated and that miRNA functions can be fully understood only by addressing TF and miRNA regulatory interactions together in a single “mixed” network. As in the case of purely transcriptional networks [10], in this mixed network several recurrent wiring patterns can be detected, called network motifs [11]–[14]. The common lore is that network motifs were selected by evolution (and are thus overrepresented in the network) to perform elementary regulatory functions. Among these motifs one of the most interesting is the miRNA-mediated feedforward loop (FFL) in which a master TF regulates a miRNA and, together with it, a set of target genes (see Figure 1). This motif, which shall be the main interest of our paper, was recently discussed in [11]–[13]. In all these papers, despite the fact that the authors used very different computational approaches, the FFL was shown to be remarkably overrepresented in the network, thus supporting the idea that it should play an important regulatory role. Depending on the sign of the transcriptional regulations, FFLs can be divided into two classes: coherent and incoherent [11], [13], [15]. In the coherent FFLs both pathways from the TF to the target have the same effect (both repressing or activating target expression), while in the incoherent ones the two pathways have opposite effects. Correspondingly one finds different expression patterns in the two cases: coexpression of miRNA and its target for incoherent FFLs and mutually exclusive expression for the coherent ones (Figure 1). This dual picture allows to better understand the complex patterns of correlated expression of miRNAs and their targets observed in experiments [1], [13], [16]. In many cases the targets show low expression in miRNA-expressing cells, suggesting coherent regulation. On the other hand, several other cases present an opposite trend, showing that miRNA repression can act in opposition to transcriptional regulation. Indeed, different degrees of expression overlap, due to different regulatory circuitries, have been related to different miRNA functions in several recent papers [1], [3], [4], [15], [17]. For example, in a coherent FFL as the one in Figure 1D, the miRNA expression is induced by an upstream TF that at the same time represses the target transcription, with the effect of enforcing mutually exclusive domains of expression as the ones observed in the fruit fly [18] or for miR-196 and its target Hoxb8 in mouse [19] and chicken [20]. In this cases the miRNA can help the transcriptional repression of a target protein that should not be expressed in a particular cell type, acting as a post-transcriptional failsafe control. Instead, an incoherent FFL (Figure 1C) can promote high target expression in miRNA-expressing cells, suggesting that miRNAs may have in this case a fine-tuning function, keeping the protein level in the correct functional range. A typical example is the regulation of the atrophin gene by the miRNA miR-8 in *Drosophila*. It was shown [21] that both a too high and a too low level of expression of the atrophin gene could be detrimental for the organism and that miR-8 is mandatory to keep the expression level exactly in the correct range.

It is by now well understood that gene espression is inherently a stochastic process [22]–[24]. This has particularly relevant effects when the number of proteins and/or messenger RNAs (mRNAs) involved is small and stochastic fluctuations may give sizeable deviations from the mean value of the final protein product. Thus, the question that naturally arises is how the cell can reconcile the fine-tuning function described above with these fluctuations. If there is only a relatively narrow protein level which is optimal, the tuning regulation must also prevent protein fluctuations outside the functional range. In fact, it has been conjectured that the incoherent FFLs that enable tuning interaction, can also have a role in buffering noise in the target expression [13], [15], [25].

The main goal of our paper is to introduce and solve analytically a stochastic model describing these incoherent FFLs in order to give a proof to this conjecture. Our results show that with respect to the simple gene activation by a TF, the introduction of a miRNA-mediated repressing pathway can significantly dampen fluctuations in the target protein output for essentially all the choices of input parameters and initial conditions. As a test of our analysis we also performed extensive numerical simulations which nicely agree with our analytical results. It is important to stress (and we shall discuss this issue in detail in the following) that this noise buffering function is actually a precise consequence of the peculiar topolgy of the FFL. In fact, in order to fine-tune the level of a target protein one would not necessarily need a FFL topology. The same result could well be obtained with an independent miRNA (not under the control of the master TF which activates the target), but this choice would lead to strong fluctuations in the target expression. In the same theoretical framework we can show that the construction of an optimal noise filter does not necessarily imply a strong repression, in agreement with the observation that the miRNA down-regulation of a target is often modest [26], [27].

## Results

### The theoretical framework

**Figure 2:**
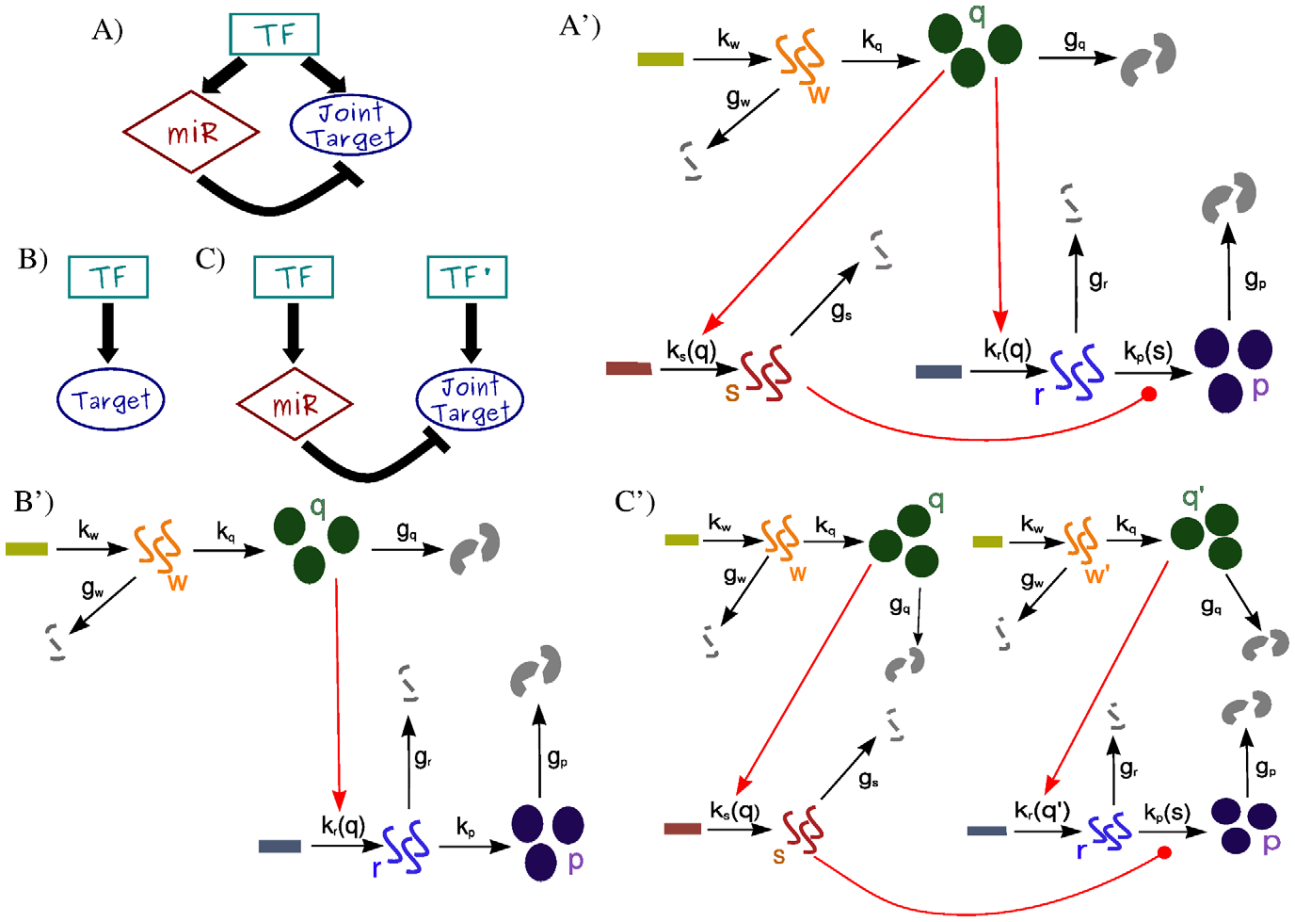
Representation of the incoherent FFL and the two circuits used for comparison. (A) A miRNA-mediated incoherent FFL that can be responsible for miRNA-target coexpression; (B) a gene activated by a TF; (C) an open circuit that leads to the same mean concentrations of the molecular species of the FFL in scheme A. (A′)(B′)(C′) Detailed representation of the modelization of the corresponding circuits. Rectangles represent DNA-genes, from which RNAs (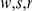) are transcribed and eventually degraded (broken lines). RNAs can be translated into proteins (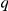 is the TF while 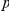 is the target protein) symbolized by circles, and proteins can be degraded (broken circles). Rates of each process (transcription, translation or degradation) are depicted along the corresponding black arrows. Regulations are represented in red, with arrows in the case of activation by TFs while rounded end lines in the case of miRNA repression. TF regulations act on rates of transcription that become functions of the amount of regulators. MiRNA regulation makes the rate of translation of the target a function of miRNA concentration.



Here we focus on the incoherent FFL in Figure 2A to present our modeling strategy. For each gene in the circuit we take into account the essential features of transcription, translation, degradation and interactions between genes in the regulatory network (detailed scheme in Figure 2A′). Accordingly, the state of the system is described by five variables 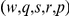 representing: 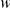 the number of mRNAs transcribed from the TF gene, 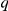 the number of TF molecules, 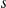 the number of miRNAs, 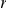 the number of mRNAs transcribed from the target gene and 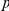 the number of target proteins. We want to explore the mean (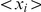) and the standard deviation (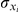) of each molecular species 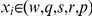 and we will show that these quantities can be obtained analitically at the steady-state. The noise strength of the species 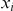 will be expressed by the coefficient of variation defined as 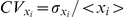. As usual in this type of models, transcriptional activation is introduced by choosing the rate of transcription of the regulated gene (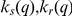 in our case) as a nonlinear increasing function of the number of TFs (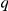) present in the cell [28]–[31]:

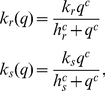
(1)where 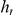 and 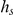 are dissociation constants, specifying the amount of TFs at which the transcription rate is half of its maximum value (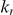 and 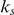 respectively). 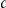 is the Hill coefficient and fixes the steepness of the activation curve.

The miRNA action can direct translational repression or destabilization of target mRNAs [32], i.e. it decreases the rate of translation or increases the rate of degradation of target mRNAs. We choose to model the effect of miRNA regulation by taking the translation rate of the target (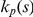) to be a repressive Hill function of the number of miRNAs (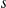):
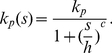
(2)


The parameter 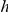 specifies the quantity of miRNAs that determines a rate of translation 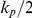, and 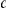 is again the Hill coefficient. For simplicity we use the same Hill coefficient 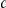 for each Hill function, but the analysis can be straigthforwardly generalized to the case of different steepnesses.

The alternative choice of a degradation rate of mRNAs as a function of miRNA concentration does not yield significantly different results, as reported in Text S1. The use of Hill functions to model regulations by miRNAs is coherent with their established catalytic action in animals [33]. A stoichiometric model has been studied in the context of sRNA regulation in bacteria [34]–[36], in which each sRNA can pair with one messenger and drive its sequestration or degradation in an irreversible fashion. A comparison with a possible stoichiometric action is shown in Text S1.

The probability of finding in our cell exactly 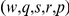 molecules at time 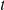 satisfies the master equation:
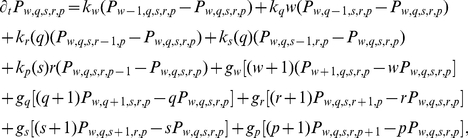
(3)


where 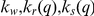 are transcription rates, 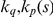 are translation rates, and 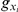 represents the degradation rate of the species 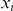.

In order to solve the master equation for 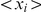 and 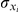 for all 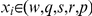 at the steady state we have to linearize Hill functions. This is by now a standard procedure [29], [30]. The idea is that at the steady state the distributions of regulators (TFs or miRNAs) have a finite width and sample only small regions of the domains of the corresponding Hill functions. We may therefore approximate Hill functions by their linearizations around the mean values of the regulators 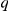 or 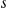 (see Text S1 for details of the linearization), ending up with:
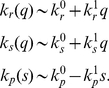
(4)


We would like to emphasize that linearizing the Hill functions does not mean to linearize the model. In fact, even with a linearized dependence on the miRNA copy number, our model keeps a nonlinear contribution in the term encoding the target translation (due to the fact that it depends on both the number of miRNAs and mRNAs). As we will see later, this nonlinearity leads to non trivial consequences.

Despite this nonlinearity, the moment generating function approach [29], [30], [37] can be succesfully used. By defining the generating function:
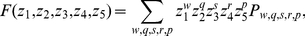
(5)and using the linearization in equation 4 we can convert equation 3 into a second-order partial differential equation:
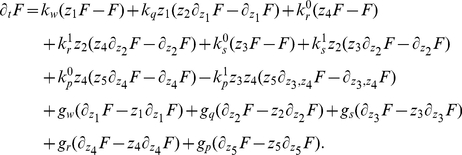
(6)


We now use the following properties of the moment generating function: 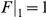; 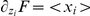; 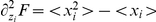 where 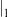 means evaluation of 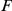 at 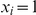 for all 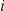. At the steady state (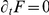) differentiation of equation 6 generates equations for successively higher moments. In particular, we are interested in 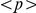 and 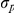 and differentiating up to the fourth moments leads to their analytical expressions (see Text S1 for details of the calculation).

Noise in protein expression is originated by the combination of two types of fluctuations: intrinsic and extrinsic ones. Intrinsic fluctuations are essentially due to the stochasticity of the gene expression process. Extrinsic ones, instead, are due to the environment. In the latter case a prominent role is played by the noise transmitted by upstream genes [38], [39]. As a matter of fact there is a certain degree of arbitrariness in the definition of extrinsic and intrinsic noise [40]. Since we focus on the target production we define “intrinsic” the noise derived from the stochastic steps of its expression (transcription, translation and degradation) and “extrinsic” the noise propagating from its regulators (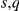) that makes the parameters (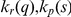) fluctuate through the Hill functions. Therefore in our model we do not have to include extrinsic noise with an arbitrary distribution as it naturally arises from the stochastic steps of production of regulators and propagates to the target gene.

### Comparison with a TF transcriptional control

**Figure 3:**
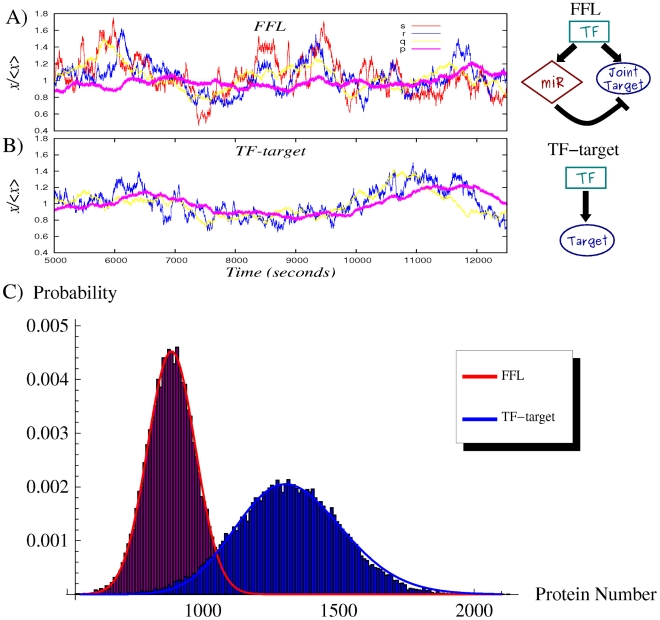
Noise properties of the FFL compared with a TF-gene linear circuit. (A) An example of simulation results for the FFL (scheme on the right or more detailed in Figure 2A′). The normalized trajectory of each molecular species is shown as a function of time after reaching the steady state. The rate of transcription of the TF is 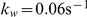 and of translation 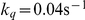. Proteins degrade with a rate 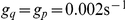, while mRNAs and miRNAs with 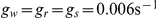. The parameters in the Hill functions of regulation (equations 1,2) are the following: the maximum rate of transcription for mRNAs is 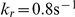, while for miRNAs is 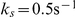; the maximum rate of translation of the target is 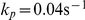; dissociation constants are 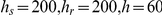; Hill coefficients are all 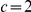, as typical biological values range from 1 (hyperbolic control) to 30 (sharp switching)[30]. (B) Time evolution in a simulation for the molecular players in the linear TF-gene cascade (scheme on the right or more detailed in Figure 2B′). Compared to the FFL case, the 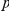 evolution is more sensitive to TF fluctuations. (C) The probability distribution of protein number for the two circuits. Histograms are the result of Gillespie simulations while continuous lines are empirical distributions (gaussian for the FFL and gamma for the TF-gene) with mean and variance predicted by the analytical model.

To show the noise buffering role of the miRNA-mediated incoherent FFL (Figure 2A) we first compare it to a simpler process: a gene activated by a TF (Figure 2B), without any post-transcriptional regulation. The strategy used to model this linear network is equivalent to the one explained in the previous section for the FFL (see Text S1 for more details) and it is presented schematically in Figure 2B′. Starting from a gene activated by a TF, in principle the gain of a new regulator implies also a new source of extrinsic noise for the target, given that the fluctuations in the number of regulators propagate to downstream genes and, as discussed in [41], the addition of extrinsic fluctuations generally increases the noise of a system. However, the peculiar structure of the FFL can overcome this problem, actually reducing noise, as was previously shown in the case of negative transcriptional auto-regulation [42]. Given that the two circuits lead to different mean values, the comparison of noise strengths in target protein will be done in terms of the coefficient of variation (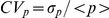). With the parameter choice explained in the caption of Figure 3, the predicted 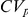 are 0.147 and 0.1 for the TF-gene cascade and the FFL respectively. Therefore the introduction of the miRNA pathway not only controls the mean value but also reduces the relative fluctuations. This effect can be clearly seen looking at the shape of the probability distributions in Figure 3C. It is rather easy to understand the origin of this noise buffering effect: any fluctuation in the concentration of TFs affects the rate of mRNA transcription, driving consequently the target protein away from its steady state, but mRNA and miRNA concentrations tend to vary in the same direction in the FFL. In this way, miRNAs can always tune the protein production against TF fluctuations. As can be seen in Figure 3A and B, there is a certain degree of correlation in the time evolution of 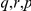 due to noise propagation, despite the overimposed higher-frequency intrinsic noise of each molecular species, but in the case of the FFL the 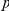 trajectory is less sensitive to 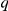 fluctuations thanks to the action of miRNAs (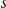). It is important to stress that this result is not affected by the Hill function linearization discussed above. In fact, the predictions of the model are in good agreement with Gillespie simulations (which keep into account the full nonlinear repressing and activating Hill functions). Moreover our results turn out to be robust with respect to parameter choice, showing a rather stable noise reduction essentially for any choice of expression and degradation constants (see Text S1 for details).

### Comparison with an open regulatory circuit

**Figure 4 pcbi-1001101-g004:**
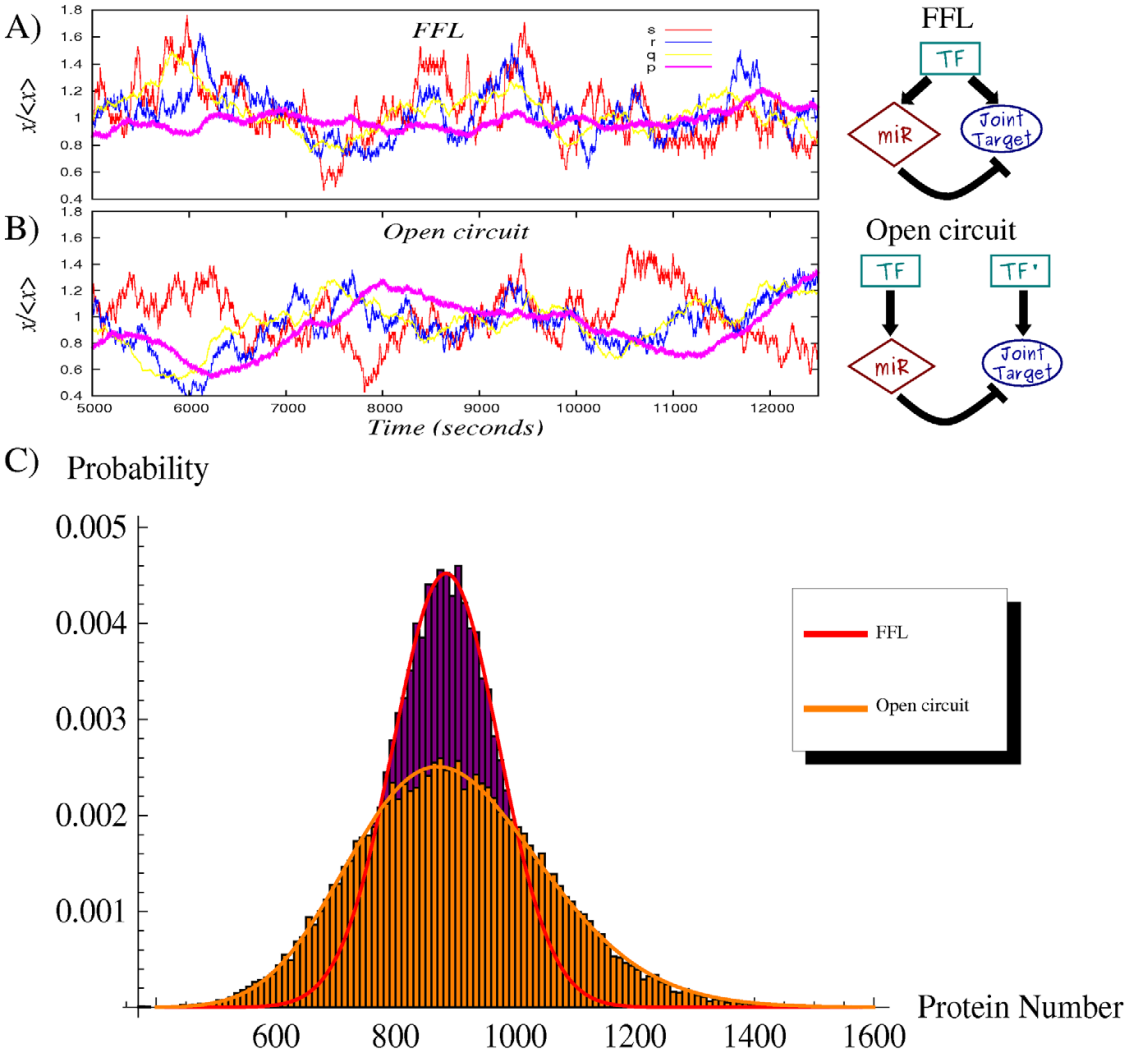
Noise properties of the FFL compared with an analogous open circuit. (A) An example of simulation results for the FFL (scheme on the right or more detailed in Figure 2A′). The parameter values are the same of Figure 3. (B) Time evolution in a simulation for the molecular players in the open circuit (scheme on the right or more detailed in Figure 2C′). The correlation between the 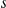 and 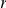 trajectories that is present in the FFL (A) is completely lost in the open circuit. As a consequence while the mean value of 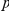 is approximately the same, its fluctuations are appreciably greater in the open circuit case. (C) The probability distribution of protein number for the two circuits. Histograms are the result of Gillespie simulations while continuous lines are empirical distributions (gaussian for the FFL and gamma for the open circuit) with mean and variance predicted by the analytical model.

The same fine-tuning of the mean target concentration achieved with a FFL could be equally obtained with an open circuit like the one in Figure 2C, where the miRNA gene is controlled by an independent TF. If the two TFs, activating the miRNA and target gene expression, have the same rate of transcription, translation and degradation of the single master TF in the FFL -as well as the other model parameters as in Figure 2A′ and C′- the stationary mean levels of the various molecular species are the same in both circuits. In particular, the mean concentration of the target protein can be fine-tuned to the same desired value by both circuits. However, while the deterministic description at the steady state is the same in the two cases (see Text S1 for details) the behaviour of fluctuations is completely different. As we shall see below, the open circuit leads to much larger fluctuations in the final product than the FFL. It is well possible that this is the reason for which FFLs have been positively selected by evolution and are presently overrepresented in the mixed TF-miRNA regulatory network. In fact, fine-tuning can be implemented advantageously only together with a fluctuation control: a precise setting of the mean value of a target protein is useless without a simultaneous damping of the stochastic fluctuations. To assess this result we used the same strategy discussed above: we solved analitically for both circuits the master equation and tested our results with a set of Gillespie simulations. Our results are shown in Figure 4: the lack of correlation between the miRNA and mRNA trajectories in the open circuit (Figure 4B) leads to much larger deviations from the mean number of proteins with respect to the FFL case. Using the same parameter values of Figure 3, the predicted 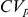 for the open circuit is 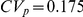, to be compared with the value 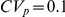 of the FFL. Different cell-to-cell variability can be clearly seen comparing the distributions of the number of target proteins for the two circuits (Figure 4C). Note that a target embedded in an open circuit has an even more noisy expression than a gene simply regulated by a TF, for which 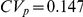.

#### Deviant effects

Stochastic equations are the natural formalism to describe a set of biochemical reactions when the number of molecules involved is small and thus fluctuations are important. As the number of molecules increases, the stochastic description smoothly converges, at least for linear systems, toward a deterministic one and stochastic equations can be substituited by ordinary differential equations (ODE). It is usually expected that even in the regime in which fluctuations cannot be neglected one could use ODE if interested only in the time evolution of the mean values. This approximation can be thought as a sort of “mean field” approach (by analogy with statistical mechanics where the mean field approximation is implemented by neglecting fluctuations). However, similarly to what happens in statistical mechanics in the proximity of a critical point, it may happen that, even at the level of mean values, the ODE description does not coincide with the (more rigorous) stochastic one. These breakdowns between the two descriptions are known as “deviant effects” [43] and are typically a consequence of nonlinear terms in the equations or of strong extrinsic fluctuations [41], [44]. In some cases these deviant effects can have relevant phenomenological consequences. This is the case of our system: although the FFL (Figure 2A,A′) and the open circuit (Figure 2C,C′) have the same deterministic description at the steady state (see Text S1 for details), the master equation approach gives a mean value of the target protein systematically lower in the FFL circuit, with respect to the same quantity in the open circuit. This is a non trivial consequence of the correlated fluctuations in the number of mRNAs and miRNAs in the FFL. This correlation between fluctuations obviously cannot be taken into account in the deterministic description and as a consequence the ODE analysis correctly describes the steady state mean number of target proteins only for the open circuit. This result can be understood by looking at the analytical expression of the mean value of 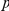:
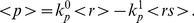
(7)


In a FFL, fluctuations of 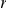 and 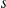 are highly correlated (Figure 3A), because the transcription rates of messengers and miRNAs depend on a shared TF. The result is that in this case 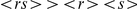. On the other hand, the production of 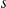 and 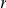 is independently regulated in an open circuit, implying that 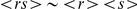. A deterministic description does not take into account fluctuations so correctly describes 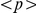 only when uncorrelated noise is averaged out without affecting mean values. In conclusion, the correlation in fluctuations introduced by the FFL topology affects the target protein mean value, establishing a systematic decrease with respect to the deterministic description. This deviant effect can be substantial and underlines the necessity of a stochastic nonlinear modeling. A fully linearized description, as for example the one proposed by [29] for post-transcriptional regulation, would not be able to show this type of effects.

### The incoherent feedforward loop is effective in reducing extrinsic fluctuations

In the previous sections we compared different regulatory circuits keeping the same amount of input noise, i.e. the same amount of fluctuations in the copy number of master TFs. Since the topology of a regulatory motif can have stronger effects on extrinsic rather than intrinsic noise [41], it would be very interesting to study how the mixed incoherent FFL behaves as a function of such extrinsic noise. As a matter of fact extrinsic and intrinsic fluctuations are generally coupled in a non-trivial way in biochemical networks [45], but we developed a strategy to control fluctuations in upstream TF expression, known to be one of the major sources of extrinsic noise in eukaryotes [39], without affecting the copy number of the molecular species in the circuit. From equation 6 we can calculate 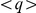 (which denotes the mean number of TFs) and its noise strength 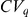:
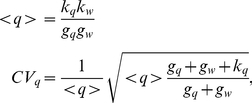
(8)where, as above, the parameters 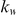 and 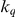 denote the rate of transcription and translation of the TF respectively, and 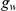 and 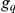 the corresponding degradation constants.

Setting the rates of degradation (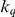 and 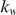) and the product 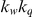 to constant values, we end up with: 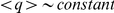 and 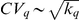. This is a well known result: fluctuations in the protein level are stronger when the rate of translation is higher [23] and can be tuned (while keeping the mean value 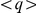 fixed) by changing the ratio 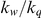 with 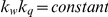. This represents a perfect theoretical setting to test the dependence of the target noise 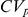 on the input noise 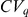, while maintaining unchanged the mean value of all the molecular species involved in the circuit.

**Figure 5 pcbi-1001101-g005:**
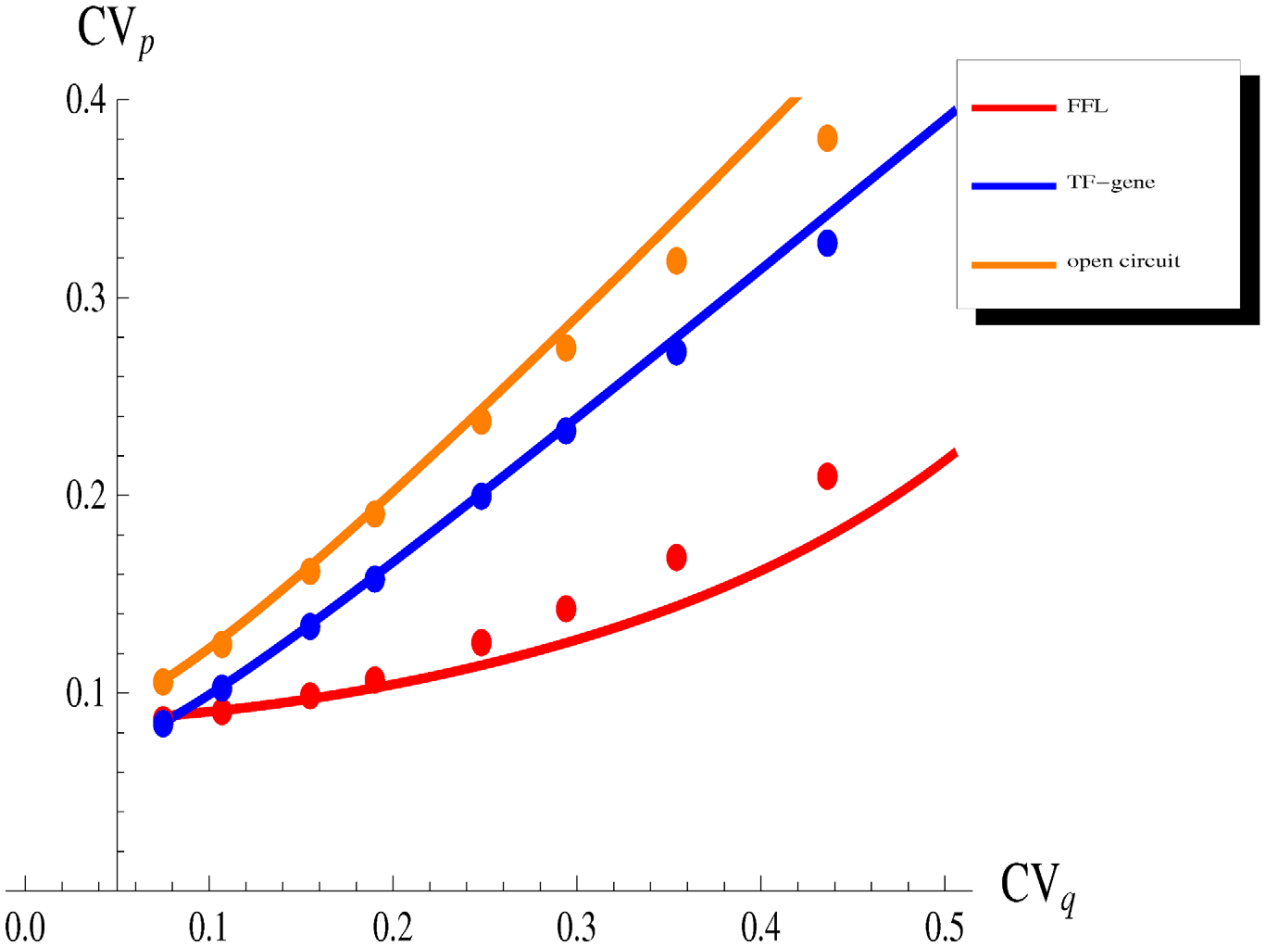
The effect of fluctuations in an upstream TF. We maintain constant the number of TFs 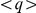, while we vary its relative fluctuations 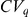, tuning the relative contribution of transcription (rate 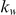) and translation (rate 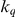). All the other parameters have the values reported in caption of Figure 3. The incoherent FFL makes the target less sensitive to fluctuations in the upstream TF. The extent of the noise reduction, with respect to the other circuits, depends on the magnitude of the TF noise, pointing out that the FFL topology is particularly effective in filtering out extrinsic fluctuations. Dots are the result of Gillespie simulations with the full nonlinear dynamics while continuous lines are analytical predictions.

We report in Figure 5 the results of such analysis for the three circuits discussed in the previous sections. While extrinsic fluctuations increase, so does the FFL's performance in filtering out noise, compared to the other circuits. Once again it is easy to understand the reason of this behaviour: the FFL architecture channels fluctuations of an upstream factor into parameters with opposite effect on the target protein, forcing them to combine destructively. Therefore the specific FFL topology seems effective in the maintenance of gene expression robustness despite noisy upstream regulators. The introduction of a miRNA pathway, building a FFL from a TF-gene cascade, really makes the difference in situations of strong upstream noise. This feature can explain why miRNAs can often be deleted without observable consequences [25], since experiments usually do not measure fluctuations and are typically performed in controlled environments, where noise is kept at minimal levels.

### Noise filtering optimization

The efficiency of the FFL in controlling the fluctuations of the target protein is a function of three main parameters: the number of master TFs (which in turn is a function of 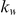 and 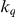), the number of miRNA copies (function of 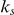 and 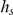) and the strength of miRNA repression (defined as 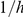). In this section we shall study the efficiency of the FFL in buffering noise as a function of each one of these three quantities, changing a corresponding parameter while keeping fixed all others. As we shall see, in all three cases the noise reduction with respect to a simple TF-target interaction (i.e. without the miRNA) shows a U-shaped profile with a well defined minimum which allows us to identify the values of the parameters which optimize the noise reduction property of the FFL. This pattern is rather robust, and only marginally depends on the way the variable of interest is tuned (for instance, by changing 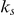 or 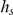 in the case of miRNA concentration). It is important to stress that in all three cases optimal noise filtering does not imply strong repression, a result which well agrees with the observation that miRNAs embedded in an incoherent FFL usually have a fine-tuning effect on the targets instead of switching them off completely. It is exactly in the intermediate region of the parameters, in which fine-tuning occurs, that we also have optimal noise reduction.

#### Optimal repression strength

**Figure 6 pcbi-1001101-g006:**
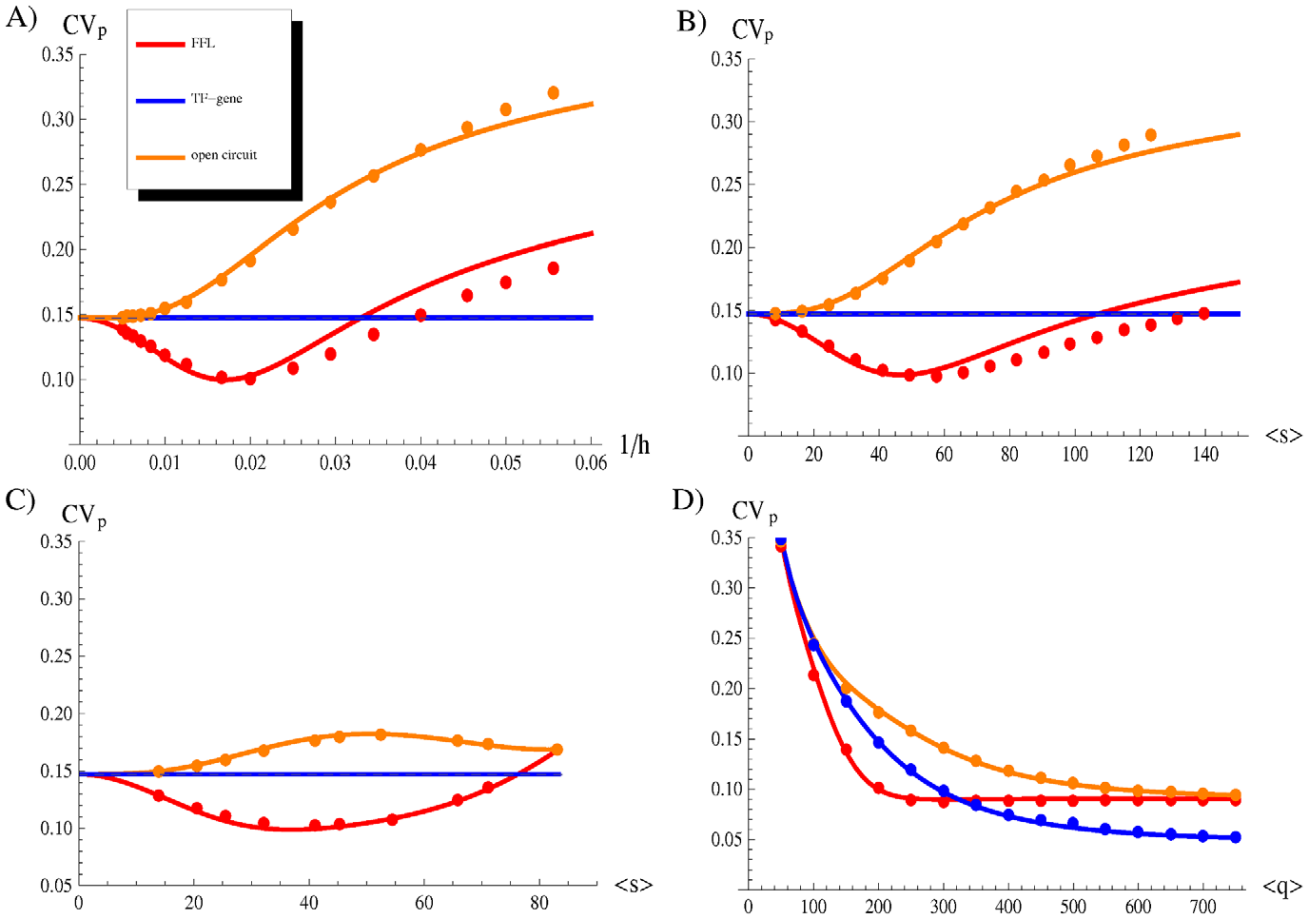
How an optimal noise filter can be built. (A) The coefficient of variation of the target protein 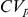 as a function of the repression strength 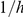. The Figure shows the presence of an optimal repression strength for which the introduction of a miRNA regulation in a FFL minimizes noise. (B) 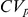 as a function of the mean number of miRNAs 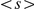. In this case 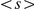 is changed through the maximum rate of transcription 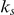 (see equation 1). (C) 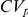 as a function of 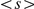, varying the dissociation constant 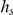. In both cases (B and C) is evident a U-shaped profile, implying an optimal noise buffering for intermediate miRNA concentrations. (D) 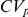 as a function of the mean number of TFs 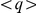. The number of TFs depends on the rate of their transcription 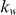 and of their translation 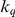. The Figure is obtained manipulating 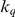, but the alternative choice of 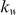 leads to equivalent results (see Text S1). For intermediate concentration of the TF, the noise control by the FFL outperforms the one of the other circuits. In each plot, dots are the result of Gillespie simulations while continuous lines are analytical predictions. The values of parameters kept constant are the same of Figure 3, however the results are quite robust with respect to their choice (see Text S1 for details).

The repression strength is defined as 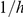 (inverse of the dissociation constant in the Hill function of equation 2). As it can be seen in Figure 6A, the FFL exhibits a noise profile with a typical U-shape and reaches an optimal value of noise reduction (measured as the difference in the noise strength 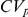 with respect to the simple TF-gene circuit) for intermediate values of repression strength. The open circuit, constrained to give the same mean value 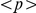, always introduces larger target fluctuations. As mentioned above, optimal noise filtering is reached for intermediate values of the repression strength and does not require strong target repression. For instance with the choice of parameter values of Figure 6, optimal noise reduction is obtained for a mean value of the target protein which is about 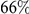 of the value obtained without the miRNA, i.e. with a simple TF-target interaction. This prediction could be experimentally tested via manipulation of the repression strength, in analogy to the work of [46] on the transcriptional autoregulatory motif. It is instructive to notice the analogies between the behaviour of the mixed FFL and that of the negative transcriptional autoregulation loop. This purely transcriptional network motif occurs ubiquitously in transcriptional regulatory networks and was recently studied in great detail [41], [47]. Also in this case, optimal noise filtering is obtained for a well defined value of the repression stength. It is easy to understand the reason of this behaviour. In a negative transcriptional autoregulation, the protein expressed from a gene inhibits its own transcription by increasing expression when protein numbers are low, while decreasing expression when protein numbers are high. Increasing the repression strength improves the circuit potential to reduce stochasticity, but at the same time decreases the copy number of mRNAs and proteins, with a rise in intrinsic fluctuations that can overcome any attenuation. Consistently, experiments show a well defined range of repression strength for which noise minimization is optimal [46].

#### Optimal miRNA concentration

Another variable which can be tuned in order to achieve optimal noise reduction is the miRNA concentration. If we keep the number of TFs constant then the miRNA concentration 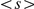 can only depend on the maximum rate of transcription of the miRNA gene (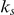) and on the affinity of its promoter to the TF (proportional to 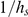, where 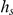 is the dissociation constant in equation 1). In Figures 6B and 6C we analyze the noise strength 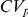 of the target protein in the FFL for different miRNA concentrations and compare it to the 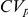 obtained with the simple TF-gene interaction and with the open circuit. Changing the miRNA concentration by varying 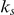 (Figure 6B) or 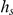 (Figure 6C) we find very similar U-shaped profiles for 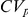. As for the previous analysis, also in this case it is possible to find an optimal miRNA concentration, and again it is such that the effect on the protein target is only a modest reduction (in this case 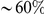 of the value obtained without the miRNA). Apart from the conserved U-shaped profile, there are rather deep differences in the noise behaviour depending on the choice of the tuning parameter. In fact, while an increase of 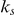 always induces an increase of 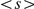, this quantity becomes insensitive to 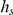 above a certain threshold. Since the number of TFs is constant in this analysis, it is clear that increasing 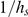 (Figure 6C) the system can reach at best the value of 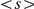 consistent with the maximum rate of transcription. At the same time a large value of 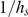 means that very few TFs are enough to support the maximum transcription rate for the miRNA gene, so fluctuations in the number of TFs become irrelevant despite the topology of the circuit. As a consequence the 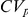 curves for the FFL and the open circuit converge to a commom value (Figure 6C). A refined experimental control of miRNA concentration through graded miRNA overexpression or silencing would permit a test of the U-shaped profile of 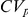 in a FFL.

#### Optimal TF concentration

The last case that we consider in this section is the effect of different TF concentrations on the noise buffering properties of the FFL. It is expected that for high TF concentrations (i.e. high values of 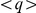) the activation functions in equations 1 reach the saturation point, making the system insensitive to variations in TF concentration. As long as the number of TFs does not fluctuates below the saturation point, the miRNA and the target gene are maximally transcribed, with no reference to the exact number of TFs. Accordingly, 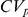 becomes asymptotically constant for large 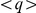 for each circuit topology (Figure 6D). The gap between the asymptotic values of the direct TF regulation and the two other circuits is due to the fact that the former does not suffer for the additional external noise due to the fluctuations in the miRNA number. On the other hand, for small values of 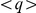 also the number of target proteins is very small as its expression is hardly activated regardless of the circuit type, with a consequent increase of the noise strength (Figure 6D). The central region is the most interesting one: this is the region in which the system is maximally sensitive to changes in TF concentration. In this regime the FFL outperforms both the simple direct regulation and the open circuit in buffering noise. Also in this case the optimal TF concentration is placed in a region corresponding to a modest reduction of 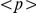, despite the miRNA repression.

#### Exploring the parameter space

**Figure 7 pcbi-1001101-g007:**
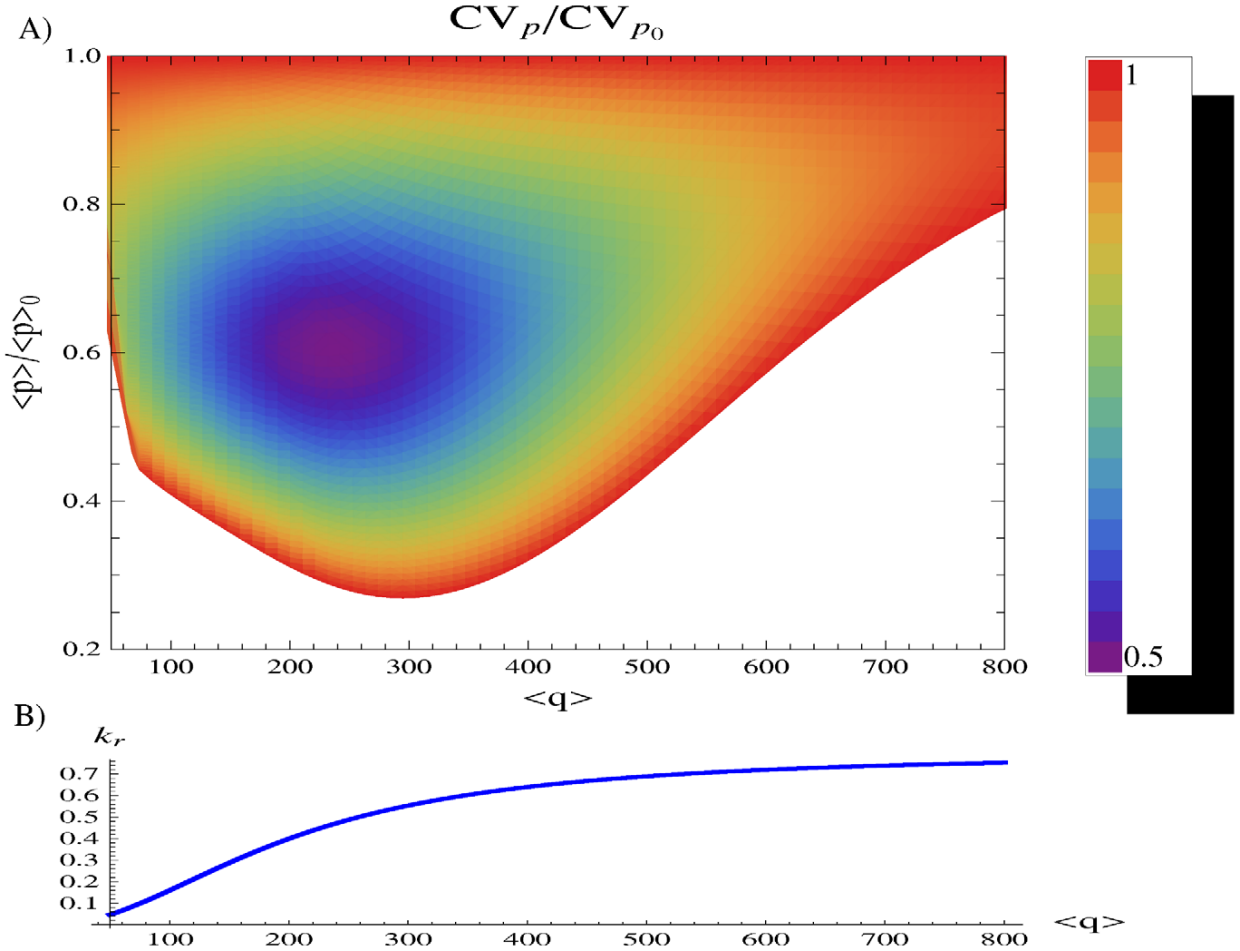
Exploring the parameter space. (A) The target noise 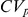, achieved with the FFL, is evaluated with respect to noise deriving from constitutive expression 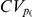 (i.e. in absence of miRNA regulation) for different mean levels of the TF 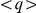 and different degrees of reduction of the target protein level 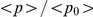 (where 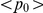 is the mean constitutive expression). The TF level is changed through its rate of translation 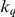 (equivalent results can be obtained changing the rate of transcription 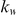), while the target level is tuned varying the repression strength. All the other parameters have the values reported in caption of Figure 3 except 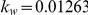 (lower than in Figure 3 to explore a more noisy situation). The region where miRNA repression leads to larger fluctuations with respect to constitutive ones is shown in white. When a noise reduction is gained the value of 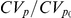 is reported with the color code explained in the legend. The best noise control is achieved with a modest suppression of target expression, around the 60% of its constitutive value. (B) The rate of transcription of the target mRNA as a function of the mean number of TFs. The noise reduction shown in the above plot can be obtained outside the saturation regime (where the slope of the activation curve tends to zero).

To give a more comprehensive insight into the relation between noise control and target repression, we finally evaluate the buffering of fluctuations (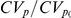) for each average number of TFs 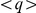 and each degree of target suppression (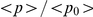), where 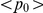 and 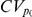 represent here the constitutive mean expression and fluctuations in absence of miRNA regulation. Results of this analysis are reported in Figure 7A. As discussed above, noise reduction can be implemented successfully in the parameter region where the target activation function (in Figure 7B) is not saturated, since this is the region where the target is sensitive to changes in TF concentration and therefore also to its fluctuations, regardless of the presence or absence of miRNA regulation. It is exactly in this region that noise buffering can be observed (compare Figures 7A and B). In particular, for each TF concentration the best noise reduction appears for a target level around 60% of its constitutive expression. In the optimal setting, noise can be remarkably reduced to about one half of its constitutive value, but the reduction remains substantial also for weaker repressions, further confirming that a strong miRNA repression is not required for noise control.

We consider the characterization of the optimal setting of miRNA-mediated incoherent FFLs for noise buffering, and the resulting U-shaped profile predicted for the noise reduction factor, as one of the major results of our analysis which, we expect, should be amenable of direct experimental validation. The fact that an optimal noise filtering is obtained with a rather modest reduction in the amount of the target protein also agrees with the recent experimental observation that miRNA down-regulation of targets is often modest [26], [27] and apparently dispensable from a functional point of view. In this respect it is tempting to conjecture that, at least for some targets of incoherent FFLs, the down-regulation could only be the side effect of an evolutionary design aiming instead to optimize noise reduction.

### Comparison with purely transcriptional incoherent feedforward loops

**Figure 8 pcbi-1001101-g008:**
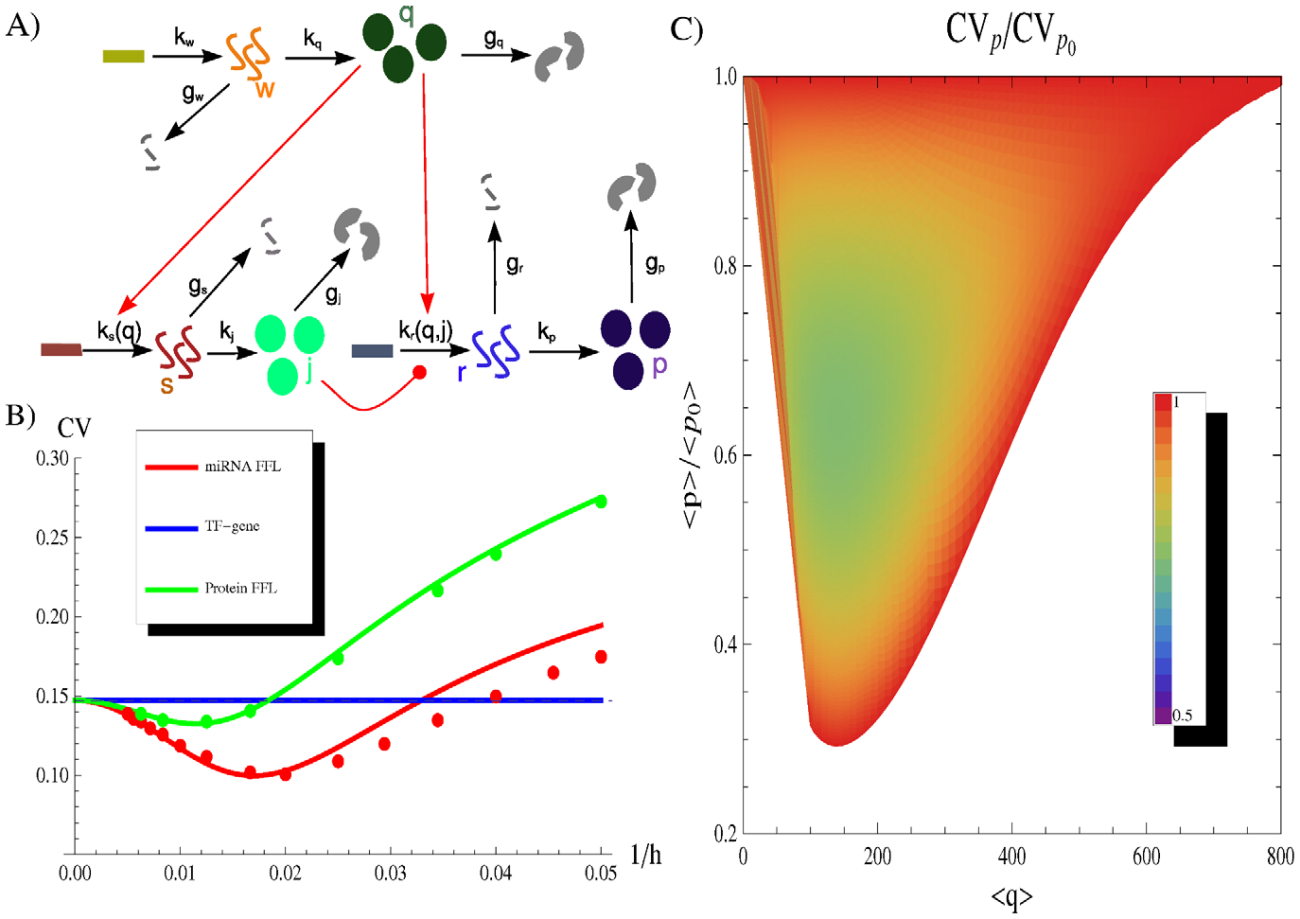
Comparison with a purely transcriptional incoherent FFL. (A) Detailed scheme of a purely transcriptional incoherent FFL. (B) The coefficient of variation of the target protein 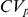 as a function of the repression strength 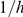 for a miRNA-mediated FFL and for its transcriptional counterpart. Thanks to the constraints imposed on parameters we can directly compare their noise-buffering efficiency with respect to a gene only activated by a TF. Both circuitries lead to a 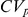 curve with a minimum for an intermediate repression strength, but the miRNA-mediated circuit appears more efficient in filtering out fluctuations. The values of parameters kept constant are the same of Figure 3. Dots are the result of Gillespie simulations with the full nonlinear dynamics while continuous lines are analytical predictions. Also in this case, analytical solutions fit nicely with simulation results. (C) The noise reduction 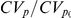, achieved with a purely transcriptional incoherent FFL, evaluated for different mean levels of the TF 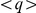 and different degrees of reduction of the target protein level 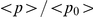. The parameter values and the color code are the same of Figure 7 so as to allow a direct comparison.

The capability of incoherent FFLs to reduce fluctuations was previously studied with simulations in the contest of purely transcriptional networks [41]. In this section we present a comparison of the noise properties of microRNA-mediated FFLs (scheme in Figure 1A′) and purely transcriptional ones (detailed scheme of reactions in Figure 8A), where the miRNA is replaced by a protein that inhibits transcription (rather than translation, as miRNAs do). The transcriptional FFL can be modeled with the same strategy explained previously for the miRNA-mediated version and analogously mean values and standard deviations of the various molecular species can be calculated analytically with the moment generating function method (see Text S1 for more details on calculations and model assumptions). In order to make an unbiased comparison of the noise properties of these two circuits, the corresponding models must be constrained to produce the same amount of target proteins. Although there is no unambiguous way to put this constraint, due to the presence of more free parameters (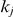 and 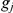) in the purely transcriptional case, a reasonable choice is to keep the shared parameters to same values (i.e repression/activation efficiencies and production/degradation rates) and choose the two additional ones to make the amount of repressor proteins 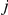 in the transcriptional case equal to the amount of miRNAs 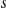 in the mixed circuit. With this choice we can evaluate the target noise 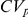 as a function of the repression strength (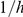) for both circuits (Figure 8B). Even though the transcriptional version can potentially reduce target fluctutions, buffering efficiency appears clearly increased by the use of miRNAs as regulators. Furthermore, a comparison of Figure 8C and Figure 7B points out that a miRNA-mediated FFL can buffer fluctuations over a wider range of conditions as well as to a greater extent. This is mainly due to the additional step of translation required for the production of proteins 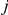 which unavoidably adds noise to the system. We would like to emphasize that the shown efficiency in noise reduction, achieved with the transcriptional FFL, should be considered as an upper bound. In fact, the constraints imposed on 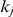 and 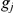 limit the translational burst size, i.e. the average number of proteins produced from a single mRNA, and this parameter crucially influences the intrinsic fluctuation amplitude of proteins 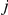 [22] (see Text S1 for details on parameter constraints). With the parameter values used in Figure 8, the translational burst size is 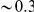, while in eukaryotes it is expected to be larger (certainly larger than one) because of the long average half-life of messenger RNAs compared to the time required for one translation round [48]. Therefore the noise added by the step of translation of proteins 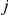 should realistically be more substantial than reported for this model setting, with harmful consequences on the noise buffering efficiency of the purely transcriptional circuit.

Moreover some peculiarities (not currently included in our model) of the mixed-motif can further explain why it can be better suited for noise buffering. Firstly, fluctuations in RNA polymerase and ribosome abundance are possible sources of extrinsic noise in gene expression [49]. These fluctuations are expected to influence unspecifically the rate of transcription and translation respectively of each gene. In a miRNA-mediated FFL the correlation between target mRNA and miRNA production, which is crucial for noise reduction, is robust to these additional sources of noise as mRNAs and miRNAs are identically affected only by global RNA polymerase fluctuations. On the other hand, in purely transcriptional FFLs the number of repressor proteins 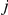 is exposed to the independent fluctuations in ribosome concentration, so the regulator-regulated correlation can be compromised with potentially negative consequences on the circuit's noise reduction efficiency.

Secondly, delays in the action of regulators (miRNA or proteins) in the indirect pathway from the master TF to the target can damage the noise buffering function (see Text S1 for a more detailed study of the impact of time delays on noise control). However, the biogenesis of miRNAs is thougth to be faster than the one of proteins, and thus miRNAs may affect the target expression with a shorter delay with respect to factors regulating nuclear events like a TF [50]. This feature should enable miRNAs to produce rapid responses, as required to counteract fluctuations.

Finally, the presence of a nucleus makes the eukaryotic cell a two-compartment system with stochastic transport channels, with consequences on gene expression noise [51]. In fact, transcriptional regulation requires an additional transport step with respect to the post-transcriptional one. In a transcriptional FFL, the repressor protein (replacing the miRNA) must return into the nucleus to act on the target. This again potentially reduces the correlation of its fluctuations with the target ones, affecting the noise buffering ability.

### Cross-talk between microRNA targets

**Figure 9:**
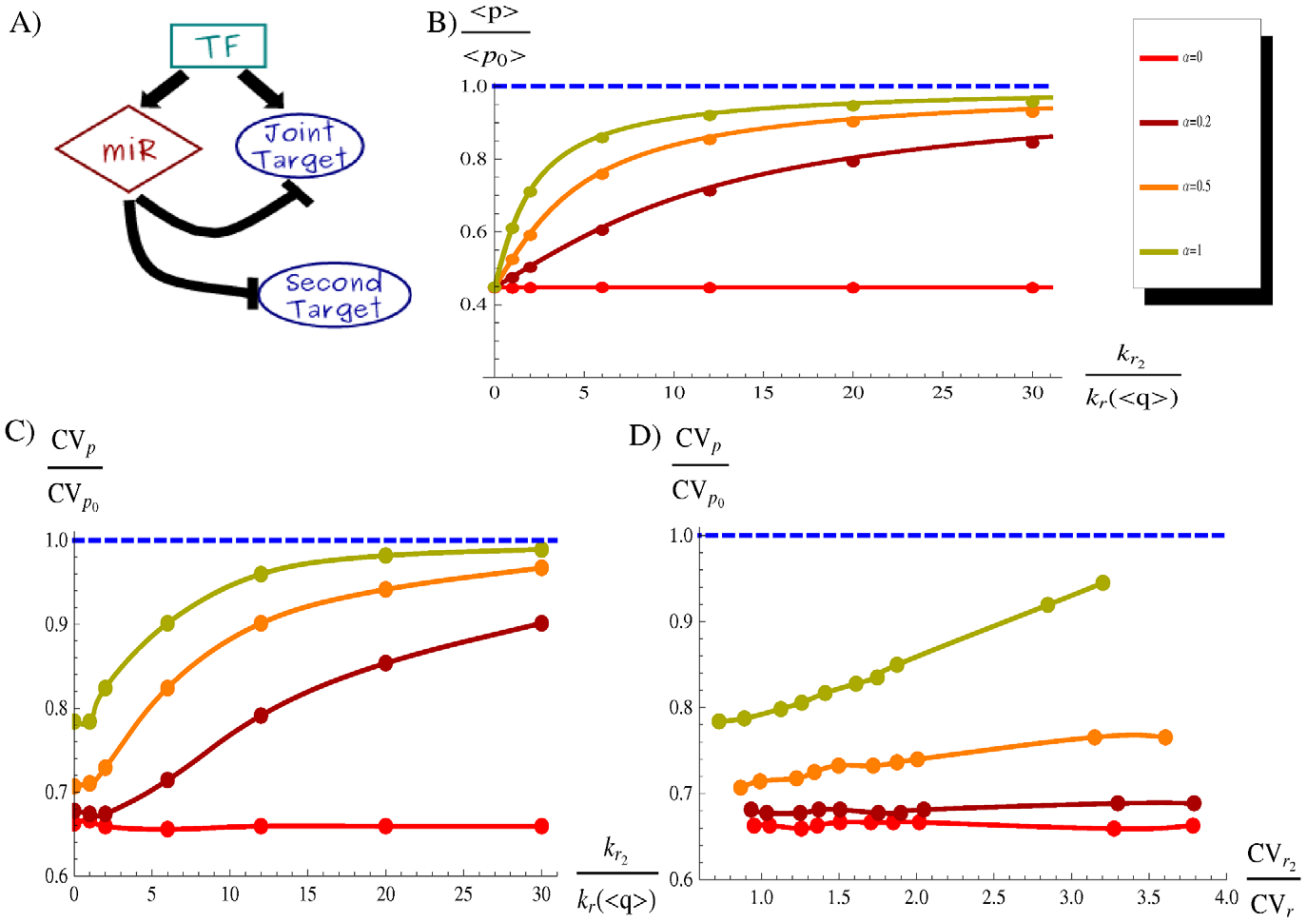
Effects of cross-talk between miRNA targets. (A) Scheme of a miRNA-mediated FFL with an additional independently transcribed target gene (second target). (B) The degree of protein downregulation 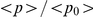 is depicted as a function of the ratio of effective transcription rates of the secondary target (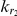) and of the FFL joint target (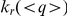), for different values of 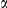. Since the rate of transcription of the joint target is a function of the TF concentration, we consider for this analysis the effective mean rate 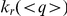 as a reference (where 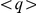 is constant as we are not tuning the TF concentration). The transcription of the second target is modeled as an independent birth-death process with birth rate 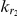. In this plot the coupling constants between targets and miRNAs are assumed equal (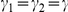) and for each 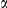 value the coupling constant 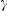 is chosen so as to start with the same amount of target proteins (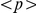) in absence of secondary targets (the complete set of parameters values is presented in Text S1). In the limit of infinite out-of-circuit target expression, the joint target protein level approaches its constitutive value if 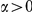, while remains constant in the ideal case of perfectly catalytic miRNA repression (red curve). Continuous lines are analytical solutions of the deterministic model (Equations 9), while dots are the result of stochastic simulations. (C) With the parameter setting of Figure 9B, the noise reduction 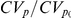 is evaluated in the same 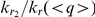 range. Dots are the result of Gillespie simulations while continuous lines come from trivial interpolations. (D) The noise reduction is evaluated as a function of the out-of-circuit mRNA fluctuations 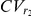, relative to the joint target fluctuations 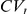. The fluctuations of the second target are modulated considering its rate of transcription as a function of an independent TF and changing the TF noise with the same strategy used for Figure 5 (see Text S1 for more details). The concentrations of the TFs activating the two targets are constrained to be equal so as to study the situation of two independent targets with the same effective transcription rate. Dots are the result of Gillespie simulations, simply interpolated with continuous lines.

A recent study pointed out that the action of a miRNA on a specific target gene expression is affected by the total number of miRNA targets and their mRNA abundance [52], a phenomenon called “dilution effect”. This is presumably a consequence of target competition for a finite intracellular pool of miRNAs. In particular, the degree of downregulation of an individual target expression is generally reduced by the presence of other transcribed target genes. A similar cross-talk between targets has been previously shown for sRNA regulation in bacteria [34] both theoretically and experimentally. Therefore, the functionality of a genetic circuit that involves miRNA regulations, as the one studied in this paper, can be influenced by the expression level of miRNA targets not embedded in the circuit. To address this issue we evaluate in this section the impact of an additional miRNA target independently transcribed (a situation depicted in Figure 9A) on the circuit ability in noise buffering.

#### Stoichiometric versus catalytic models of miRNA action

The model used so far for miRNA regulation was based on the hypothesis of perfectly catalytic action. The rate of translation of target mRNAs was assumed to be a nonlinear decreasing function of miRNA concentration, neglecting the details of mRNA-miRNA physical coupling with the implicit assumption that the downregulation process does not affect the available miRNA pool. A perfectly catalytic action does not predict any competition effect among multiple targets at equilibrium, since each target can only sense the available number of miRNAs without altering it. On the other hand, a stoichiometric model has been proposed in the context of sRNA regulation in bacteria [34]–[36], in which each sRNA can pair with one messenger leading to mutual degradation. In this latter case the expression of a secondary target can capture a significant portion of the sRNAs, with a resulting decrease in the average repression acting on the first target. The nature of miRNA regulation is presumably somewhere in between these two extreme possibilities, although usually generically referred to as catalytic. In this view, in order to address the effect of target cross-talk on miRNA-mediated FFLs, we consider a deterministic model (introduced previously in [34]) that explicitely takes into account the physical coupling of miRNAs and target mRNAs and the catalytic/stoichiometric nature of this coupling. While the full detailed model is presented in Text S1, the effective equations describing the dynamics of the mean number of miRNAs 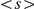, mRNAs 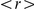 of the target in the FFL and mRNAs 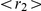 of the secondary miRNA target are:
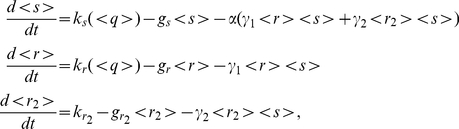
(9)where 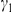 and 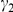 describe the probability of miRNA-mRNA coupling for the target in the FFL and the secondary target respectively, while 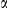 is the probability (assumed equal for both targets) that a degradation event of a mRNA, induced by a miRNA, is accompained by the degradation of the miRNA itself. The limit 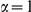 describes a stoichiometric mode of action, while the opposite situation of 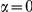 represents a perfectly catalytic mode in which the rate of mRNA degradation is a linear function of the number of miRNAs.

The corresponding stochastic model, of which equations 9 describe the mean-field limit, cannot be solved analytically starting from the master equation, therefore noise properties will be examined in the following with simulations only.

#### Dilution effect

In the first place we evaluate the dependence of the target protein downregulation on the expression rate of the secondary target, starting from the model described by Equations 9. The dilution effect is shown in Figure 9B for different values of 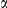: the downregulation exerted on the FFL target depends on the rate of expression of the secondary target, in line with the observed inverse correlation between target abundance and mean downregulation in higher eukaryotes [52] and in bacteria [34]. Similar results can be obtained by varying the coupling constant 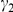 with respect to 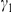 (as reported in [34]). Therefore, the noise buffering function and the optimality criteria discussed in previous sections could be compromised in the presence of many or highly transcribed independent miRNA targets. This issue will be addressed in details in the following section.

As expected, a perfectly catalytic mode does not feel the effect of secondary mRNA targets (red line in Figure 9B), while the stoichiometric mechanism is the most sensitive (green line in Figure 9B). This result suggests that a catalytic mode (at least approximately), like the miRNA one, can allow a larger proliferation of the number of targets while limiting the effects of their cross-talk.

#### Consequences of dilution effect and secondary target fluctuations on noise buffering

Since a high level of expression of secondary targets can determine a decrease of the average downregulation, it can potentially reduce the FFL ability in filtering out target fluctuations. In fact, also the noise reduction 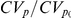 (where 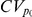 is the constitutive noise in absence of miRNA) is a function of the additional target expression, as shown in Figure 9C. As the expression of the out-of-circuit target increases, its messengers are able to capture more and more miRNAs and the efficiency in noise reduction is gradually compromised. Finally the FFL target fluctuations 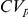 approach the constitutive ones 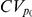 when the messengers of the FFL target become a small fraction of the total miRNA targets. The robustness of the circuit functioning with respect to the dilution effect is again dependent on the repression mode (that changes with 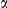). Moreover, as discussed in Text S1, different modes (stoichiometric/catalytic) of miRNA action have a different potential in reducing fluctuations: even in absence of secondary targets, where models with different 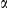 have been constrained to produce the same amount of target protein, the noise buffering efficiency decreases with 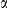 (Figure 9C). This observation highlights that the level of miRNA ability to avoid mutual degradation while targeting a mRNA can play a role in the optimization of fluctuation counteracting, besides conferring stability with respect to target cross-talk.

While the corruption of the noise-buffering ability seems mainly due to the increase in the mean level of secondary messengers, there is another more subtle cause that gives a contribution: the uncorrelated fluctuations of secondary messengers. Since the secondary target is independently transcribed (not under the control of the master TF activating the miRNA gene) its fluctuations are expected to be completely uncorrelated with the miRNA ones, implying a random sequestration of miRNAs. To disentagle this contribution from the dilution effect, we studied the case of a secondary target transcribed at the same effective rate of the FFL target, but with different levels of fluctuations (see Figure 9D). In the case of equal transcription rates the dilution effect has a negligible impact on the noise buffering activity of the circuit (see Figure 9C), nevertheless the level of noise reduction (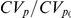) is progressively reduced as the second target concentration becomes more and more noisy, as reported in Figure 9D. This effect seems especially relevant for a hypothetically stoichiometric miRNA repression. Therefore, the noise level of additional targets is a variable that must be taken into account in evaluating the cross-talk effect on the noise-buffering efficiency of the circuit. Although the FFLs are overrepresented in the mixed network [11]–[14], a single microRNAs can downregulate hundreds of target genes and consequently not every target is expected to be under the control of the same TF regulating the miRNA gene (see Text S1 for a more detailed discussion). Therefore, even though most motif function analysis are carried out looking at the motif operating in isolation, we have shown that the presence of additional miRNA targets in the network can alter the functioning of a miRNA-mediated motif. In fact, the efficiency of miRNA-mediated FFLs as noise controllers should be considered contest-dependent. While this circuit seems properly designed to filter out fluctuations when the miRNA-target interaction is specific or secondary targets are poorly transcribed, cell types or conditions that require a high expression of out-of-circuit miRNA targets can significantly corrupt this circuit property. Besides the understanding of the function of endogenous miRNA-mediated FFLs, this analysis of target cross-talk effects can be a useful warning for the growing field of synthetic biology [53]: the implementation of genetic circuits incorporating small RNA regulations for specific scopes must take into account the sRNA specificity and the level of expression (and fluctuations) of eventual other targets.

## Discussion

### Experimental and bioinformatic evidences of the relevance of miRNA mediated FFLs in gene regulation

Few cases of incoherent miRNA-mediated FFLs have been experimentally verified until now: a case involving c-Myc/E2F1 regulation [54] and more recently a miR-7 mediated FFL in *Drosophila* [50]. As a matter of fact, miR-7 has indeed been found to be essential to buffer external fluctuations, providing robustness to the eye developmental program. The fact that miR-7 is interlocked in an incoherent FFL provides a first hint that our model can be biologically relevant.

On the purely computational side, it is interesting to notice that in [11] it was shown that the typical targets of these FFLs are not randomly distributed but are instead remarkably enriched in TFs. These are the typical genes for which a control of stochastic fluctuations should be expected: the noise in a regulator expression propagates to all its targets, affecting the reliability of signal transmission in the downstream network.

Finally, a significant enrichment in oncogenes within the components of the FFLs was also observed [11]. The mentioned FFL containing c-Myc/E2F1 is just an example [8]. In view of the emerging idea that non-genetic heterogenetity, due to stochastic noise, contributes to tumor progression [55] and affects apoptotic signal response [56], the role of miRNA-mediated FFLs in reducing fluctuations can explain why they are often involved in cancer-related pathways.

### Concluding remarks

The type of regulatory action which a miRNA exerts on its targets can be rather well understood looking at the degree of coexpression with the targets [1], [3], [4], [15], [17]. In particular, an incoherent mixed-FFL implies a high level of miRNA-target coexpression, so it is suitable to implement a fine-tuning interaction. The target is not switched off by miRNA repression, rather its mean level is adjusted post-transcriptionally to the desired value. However, many cells can have a protein concentration far from the finely controlled mean value, if strong fluctuations are allowed. Hence, a noise buffering mechanism can be crucial at the level of single cells, and a fine-tuning interaction will be effective for a large part of the cell population only if coupled with a noise control. Some authors proposed the conjecture that the incoherent mixed-FFL can actually have a role in noise buffering [13], [15], [25] and biological evidences that miRNAs can effectively be used as expression-buffers have been recently found [25], [50]. From this point of view the miRNA-target interactions classified as neutral [17], as the mean level of the target only changes inside its functional range by the presence/absence of miRNAs, actually could have been selected by evolution to prevent potentially harmful fluctuations. In this paper we demonstrated, through stochastic modeling and simulations, that the incoherent mixed-FFL has the right characteristics to reduce fluctuations, giving a proof to the previously proposed intuitive conjecture and supplying the lacking quantitative description. In particular, we showed that this circuit filters out the noise that is propagating from the master TF, giving robustness to the target gene expression in presence of noisy upstream factors. Furthermore, our theoretical description led to the prediction that there is a value of the miRNA repression strength for which the noise filtering is optimal. A maximum of target-noise attenuation appears likewise varying the miRNA concentration or the TF concentration and this robust prediction could be tested experimentally. In all cases the implementation of the best noise filter does not imply a strong suppression of the target protein expression, coherently with a fine-tuning function and in agreement with the observation that the miRNA down-regulation of a target is often modest [26], [27].

Our paper presents the first model explicitly built on the mixed version of the FFL. From a theoretical point of view, we addressed the detailed master equation describing the system (without neglecting the dynamics of mRNA), instead of the approximate Langevin description, and we were able to apply the moment generating function approach despite the presence of nonlinear terms that can give rise to deviant effects. This approach allowed us to take into account extrinsic fluctuations as the noise propagating from upstream genes, without an arbitrary definition of the extrinsic noise distribution. This strategy can be naturally extended to other circuits in the mixed network to test their potential role in the control of stochasticity.

Furthermore, we compared, in terms of noise buffering ability, miRNA-mediated FFLs with their purely transcriptional counterparts, where the miRNA is replaced by a protein that inhibits transcription rather than translation. This comparison shows that a miRNA regulator can be better suited for the noise buffering purpose.

Finally, we tryed to overcome the limitations in the analysis that can arise from considering a genetic circuit as operating in isolation. In this perspective, we evaluated the impact that the recently discovered dilution effect [34], [52] can have on the noise buffering function of miRNA-mediated incoherent FFLs. More specifically, we showed than an efficient noise control requires the minimization of the number of miRNA target sites on out-of-circuit genes, especially if highly expressed or strongly fluctuating in the mRNA level.

The hypothesis of a role of miRNAs in noise buffering can shed new light on peculiar characteristics of miRNA regulation. As discussed in [25] and [50], it can explain why miRNAs are often highly conserved, controlling key steps in development, but in many cases they can be deleted with little phenotypic consequences. On the evolutionary side, the origin of vertebrate complexity seems to correspond to the huge expansion of non-coding RNA inventory (including miRNAs) [57]. This can suggest a further reasoning: the morphological complexity requires a high degree of signaling precision, with a strict control of stochasticity, and miRNA regulation can satisfy these requirements if embedded in an appropriate circuit, as we showed for the ubiquitous miRNA-mediated FFL.

## Methods

Simulations were implemented by using Gillespie's first reaction algorithm [58]. The reactions simulated were those presented in schemes 2A′,B′,C′ and 8A. Reactions that depend on a regulator were allowed to have as rates the corresponding full nonlinear Hill functions. All the results are at steady state, which is assumed to be reached when the deterministic evolution of the system in analysis is at a distance from the steady state (its asymptotic value) smaller than its 0.05% (see Text S1 for details). For the parameter set used for Figures 3-9 the steady state was assumed at 5000 seconds, around 14 times the protein half-life. Each data point or histogram is the result of 100000 trials.

## Supporting Information

Text S1 Details on the theoretical model, supplementary analysis, and simulations.
(0.75 MB PDF)
