# Supplementary material for: The Role of Incoherent MicroRNA-Mediated Feedforward Loops in Noise Buffering
Source: PLoS Comput Biol. 2011 Mar 10;7(3):e1001101. doi: 10.1371/journal.pcbi.1001101 (PMC3053320; doi:10.1371/journal.pcbi.1001101)
Supplement: Text S1 — Details on the theoretical model, supplementary analysis, and simulations. (0.75 MB PDF) [file pcbi.1001101.s001.pdf]

# **Text S1:** Supporting Information for the article "The role of incoherent microRNA-mediated feedforward loops in noise buffering"

M Osella, C Bosia, D Corá and M Caselle

## Contents

**1 Deterministic models** **3**
- 1.1 The TF-gene linear circuit . . . 3
- 1.2 The FFL . . . 4
- 1.3 The open circuit . . . 6

**2 Stochastic Models** **7**
- 2.1 Linearization of Hill functions . . . 7
- 2.2 The TF-gene linear circuit . . . 8
  - 2.2.1 Moments of the distribution . . . 9
- 2.3 The FFL . . . 9
- 2.4 The open circuit . . . 10
- 2.5 Numerical definition of the steady-state time-threshold . . . 11
- 2.6 Robustness of our results with respect to changes in the values of the input parameters . 12
  - 2.6.1 Constraints imposed on the FFLs by the requirement of sensitivity to changes in the master TF concentration. . . . 12
  - 2.6.2 Target and miRNA genes differentially expressed . . . 13
  - 2.6.3 mRNAs and miRNAs with different stability . . . 13
  - 2.6.4 Optimal TF concentration tuning $k_w$ instead of $k_q$ . . . 13
  - 2.6.5 Results for another set of parameters . . . 14
- 2.7 Testing the effect of Hill function linearization . . . 15

**3 MiRNA-mediated promotion of mRNA degradation** **17**
- 3.1 Deterministic model . . . 18
- 3.2 Comparison with miRNA-mediated repression of mRNA translation . . . 18

**4 Stoichiometric mechanism of repression** **19**
- 4.1 Deterministic model . . . 20
- 4.2 Comparison with catalytic repression . . . 21

**5 Purely transcriptional incoherent FFLs** **22**
- 5.1 Stochastic model . . . 22
- 5.2 Constraints on parameters for a comparison with miRNA-mediated FFLs . . . 24

**6 Cross-talk between miRNA targets** **25**
6.1 Stoichiometric vs catalytic model of miRNA action . . . . . . . . . . . . . . . . . . . . . . 25
6.2 Details on the model setting . . . . . . . . . . . . . . . . . . . . . . . . . . . . . . . . . . . 26
6.2.1 Setting for Figure 9 B . . . . . . . . . . . . . . . . . . . . . . . . . . . . . . . . . . 26
6.2.2 Setting for Figure 9 C . . . . . . . . . . . . . . . . . . . . . . . . . . . . . . . . . . 27
6.2.3 Setting for Figure 9 D . . . . . . . . . . . . . . . . . . . . . . . . . . . . . . . . . . 27

**7 Noise reduction and signaling sensitivity** **27**

**8 Effects of possible delays in miRNA production.** **29**

**9 Bioinformatical analysis of miRNAs involved in FFLs in the human mixed network.** **30**

# 1 Deterministic models

In this section we consider the mean field descriptions at the steady state of the three networks in analysis: a TF-gene linear circuit without any post-transcriptional control, an incoherent miRNA-mediated FFL and an open circuit. As we will show the open circuit is built in order to have the same mean levels of molecular species (in particular of target proteins) that are obtained with the FFL. This feature makes the open circuit a suitable null model in order to disentangle the topology contribution to noise buffering. In this section the variables that describe the state of the system ( $\{w, q, r, s, p\}$ for the FFL) are continuous variables.

## 1.1 The TF-gene linear circuit

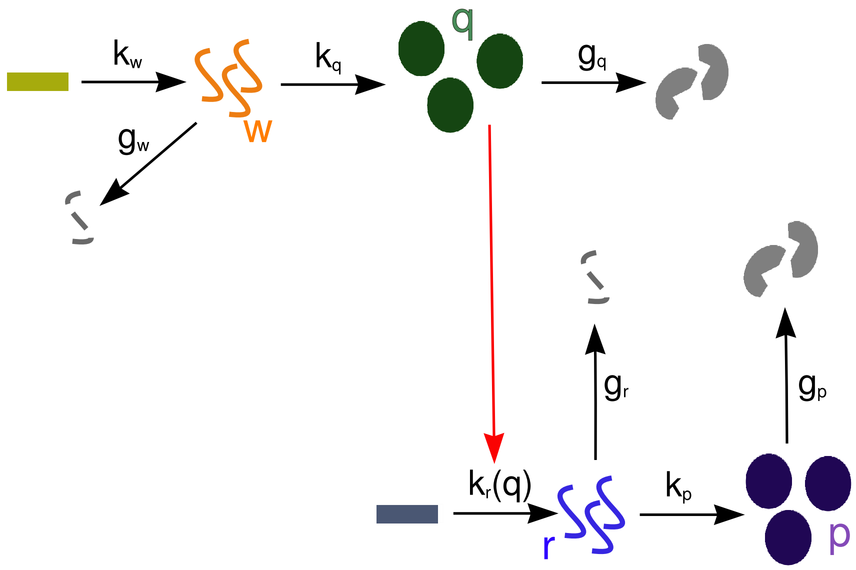


**Figure 1.** Scheme of a TF-gene linear circuit. Rectangles represent DNA-genes, from which RNAs $(w, r)$ are transcribed and eventually degraded (broken lines). RNAs can be translated into proteins ($q$ is the copy number of TFs while $p$ of target proteins) symbolized by circles, and proteins can be degraded (broken circles). Rates of each process (transcription, translation or degradation) are depicted along the corresponding black arrows. Regulations are represented in red, with arrows indicating activation by TFs.

A deterministic description of the scheme in Fig.1 is given by the equations:

$$\begin{aligned}\frac{dw}{dt} &= k_w - g_w w \\ \frac{dq}{dt} &= k_q w - g_q q \\ \frac{dr}{dt} &= k_r(q) - g_r r \\ \frac{dp}{dt} &= k_p r - g_p p, \end{aligned} \tag{1}$$

where the rate of transcription of the target mRNAs $(r)$ is a Hill function of the number of TFs $(q)$:

$$k_r(q) = \frac{k_r q^c}{h_r^c + q^c}. \tag{2}$$

At the steady state, where $dx_i/dt = 0 \; \forall x_i \in \{w, q, r, p\}$, the system of equations can be solved, ending up with:

$$\begin{aligned} w_{ss} &= \frac{k_w}{g_w} \\ q_{ss} &= \frac{k_q k_w}{g_q g_w} \\ r_{ss} &= \frac{k_q^2 k_r k_w^2}{g_r (g_q^2 g_w^2 h_r^2 + k_q^2 k_w^2)} \\ p_{ss} &= \frac{k_p k_q^2 k r k_w^2}{g_p g_r (g_q^2 g_w^2 h_r^2 + k_q^2 k_w^2)}, \end{aligned} \tag{3}$$

where the subscript $ss$ indicates the evaluation at the steady state and we assumed $c = 2$.

## 1.2 The FFL

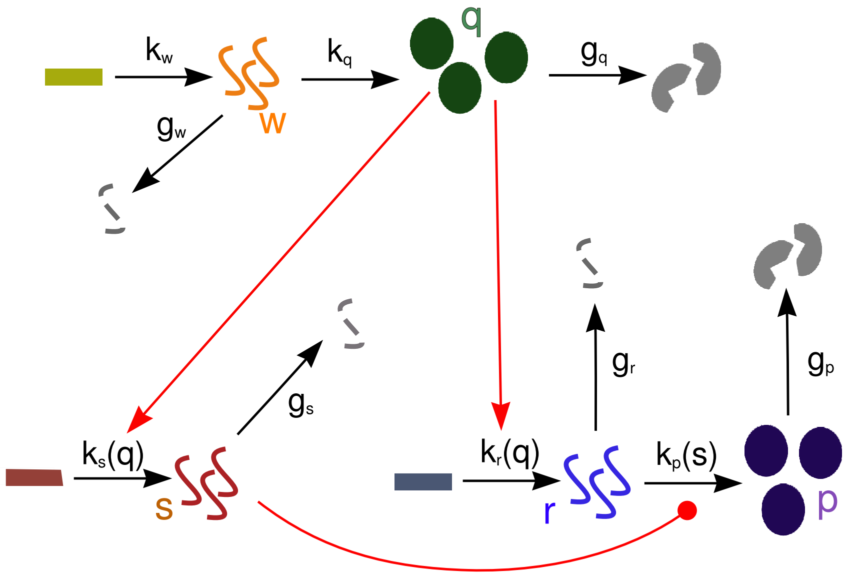


**Figure 2.** Scheme of a miRNA-mediated incoherent FFL. Notations are the same of Fig.1. The only difference with respect to Fig.1 is the presence of the miRNA gene, activated by the TF (red arrow). MiRNA regulation of the target (red rounded end line) makes its rate of translation a function of miRNA concentration.

A deterministic description of the scheme in Fig.2 is given by the equations:

$$\begin{aligned}
\frac{dw}{dt} &= k_w - g_w w \\
\frac{dq}{dt} &= k_q w - g_q q \\
\frac{ds}{dt} &= k_s(q) - g_s s \\
\frac{dr}{dt} &= k_r(q) - g_r r \\
\frac{dp}{dt} &= k_p(s) r - g_p p. \qquad (4)
\end{aligned}$$

The transcription rates of the miRNA gene and of the target gene are Hill functions of the number of TFs ($q$), while the translation rate of the target gene is a repressive Hill function of the number of miRNAs ($s$):

$$\begin{aligned}
k_r(q) &= \frac{k_r q^c}{h_r^c + q^c} \\
k_s(q) &= \frac{k_s q^c}{h_s^c + q^c} \\
k_p(s) &= \frac{k_p}{1 + (\frac{s}{h})^c}. \qquad (5)
\end{aligned}$$

For simplicity we use the same Hill coefficient $c$ for each Hill function, but the analysis can be straigthforwardly generalized to the case of Hill functions with different steepness.
At the steady state, where $dx_i/dt = 0 \; \forall x_i \in \{w, q, s, r, p\}$, the system of Eqs.4 can be solved (we assume again $c = 2$), ending up with:

$$\begin{aligned}
w_{ss} &= \frac{k_w}{g_w} \\
q_{ss} &= \frac{k_q k_w}{g_q g_w} \\
s_{ss} &= \frac{k_q^2 k_s k_w^2}{g_s(g_q^2 g_w^2 h_s^2 + k_q^2 k_w^2)} \\
r_{ss} &= \frac{k_q^2 k_r k_w^2}{g_r(g_q^2 g_w^2 h_r^2 + k_q^2 k_w^2)} \\
p_{ss} &= \frac{h^2 k_p k_q^2 k_r k_w^2}{g_p g_r (g_q^2 g_w^2 h_r^2 + k_q^2 k_w^2)\left(h^2 + \frac{k_q^4 k_s^2 k_w^4}{g_s^2 (g_q^2 g_w^2 h_s^2 + k_q^2 k_w^2)^2}\right)}. \qquad (6)
\end{aligned}$$

The results for $w_{ss}, q_{ss}, r_{ss}$ coincide with the corresponding ones of the TF-gene linear cascade (see Eqs.3). $s_{ss}$ and $r_{ss}$ have the same functional dependence on the input parameters (except for the obvious substitutions $k_r \leftrightarrow k_s$, $g_r \leftrightarrow g_s$ and $h_r \leftrightarrow h_s$) as their expression depends on the amount of TFs in the same way. On the contrary $p$ has a different expression with respect to the linear circuit TF-gene, as in this case additional terms, related to miRNA repression, appear.

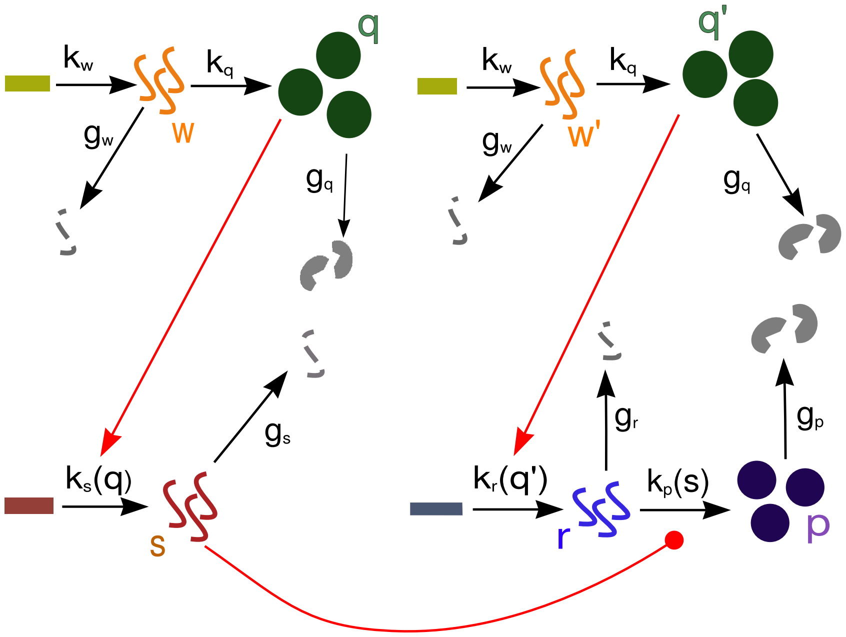


**Figure 3.** Scheme of an open circuit that can lead to the same mean concentrations of molecular species of the FFL. The notation is the same of Fig.1. Unlike the FFL case, here the miRNA and the target gene are activated by two indipendent TFs, present in copy numbers $q$ and $q'$.

## 1.3 The open circuit

A deterministic description of the scheme in Fig.3 is given by the equations:

$$
\begin{aligned}
\frac{dw}{dt} &= k_w - g_w w \\
\frac{dq}{dt} &= k_q w - g_q q \\
\frac{dw'}{dt} &= k_w - g_w w' \\
\frac{dq'}{dt} &= k_q w' - g_q q' \\
\frac{ds}{dt} &= k_s(q) - g_s s \\
\frac{dr}{dt} &= k_r(q') - g_r r \\
\frac{dp}{dt} &= k_p(s) r - g_p p.
\end{aligned} \tag{7}
$$

The presence of two independent TFs (copy numbers $q$ and $q'$) that regulate respectively the transcription of $s$ and $r$ does not change the expression of $p_{ss}$ previously obtained in the FFL case, as long as their rate of transcription, translation and degradation are the same of that of the single TF in the FFL and assuming that the Hill functions of activation of the target gene and the miRNA gene are exactly the same. The solutions of Eqs.7 at equilibrium (with $c = 2$) are:

$$
\begin{aligned}
w_{ss} &= w'_{ss} = \frac{k_w}{g_w} \\
q_{ss} &= q'_{ss} = \frac{k_q k_w}{g_q g_w} \\
s_{ss} &= \frac{k_q^2 k_s k_w^2}{g_s(g_q^2 g_w^2 h_s^2 + k_q^2 k_w^2)} \\
r_{ss} &= \frac{k_q^2 k_r k_w^2}{g_r(g_q^2 g_w^2 h_r^2 + k_q^2 k_w^2)} \\
p_{ss} &= \frac{h^2 k_p k_q^2 k_r k_w^2}{g_p g_r(g_q^2 g_w^2 h_r^2 + k_q^2 k_w^2)\left(h^2 + \frac{k_q^4 k_s^2 k_w^4}{g_s^2(g_q^2 g_w^2 h_s^2 + k_q^2 k_w^2)^2}\right)}.
\end{aligned} \tag{8}
$$

Therefore, this open circuit allows the same setting of the concentration of target proteins. As mentioned above, this feature makes the open circuit a good null model for comparison with the FFL: as the mean field description is the same, any difference between the two will be due to stochastic fluctuations.

# 2 Stochastic Models

We present the master equations for the three circuits discussed in the previous section (Fig.1,2,3), keeping into account the discrete and stochastic nature of chemical reactions. The strategy to find the expression of $< p >$ and $CV_p$ at the steady state is the method of the moment generating function (as discussed in the main text). In this section the variables describing the system ($\{w, q, r, s, p\}$ for the FFL) are discrete and represent the actual number of molecules at a specific time. The notation $< x_i >$ indicates the mean value at the steady state for the variable $x_i$.

## 2.1 Linearization of Hill functions

As a first step, following [1,2] we linearize Hill functions in Eqs.5. This is a commonly used approximation [1,2] and it is based on the idea that at the steady state the distributions of regulators (TFs or miRNAs) have a finite width and sample only small regions of the domains of the corresponding Hill functions. We may therefore approximate Hill functions by their linearizations about mean values of the regulators $q$ or $s$:

$$
\begin{aligned}
k_r(q) &\sim k_r(q)|_{<q>} + \partial_q k_r(q)|_{<q>}(q- < q >) \\
k_s(q) &\sim k_s(q)|_{<q>} + \partial_q k_s(q)|_{<q>}(q- < q >) \\
k_p(s) &\sim k_p(s)|_{<s>} + \partial_s k_p(s)|_{<s>}(s- < s >).
\end{aligned} \tag{9}
$$

Defining:

$$
\begin{aligned}
k_r^0 &= k_r(q)|_{<q>} - \partial_q k_r(q)|_{<q>} < q > \\
k_r^1 &= \partial_q k_r(q)|_{<q>} \\
k_s^0 &= k_s(q)|_{<q>} - \partial_q k_s(q)|_{<q>} < q > \\
k_s^1 &= \partial_q k_s(q)|_{<q>} \\
k_p^0 &= k_p(s)|_{<s>} - \partial_s k_p(s)|_{<s>} < s > \\
k_p^1 &= -\partial_s k_p(s)|_{<s>}, \qquad (10)
\end{aligned}
$$

and substituting in Eqs.9 we obtain Eqs.4 of the main text:

$$
\begin{aligned}
k_r(q) &\sim k_r^0 + k_r^1 q \\
k_s(q) &\sim k_s^0 + k_s^1 q \\
k_p(s) &\sim k_p^0 - k_p^1 s. \qquad (11)
\end{aligned}
$$

## 2.2 The TF-gene linear circuit

The master equation that describes the circuit in the scheme of Fig. 1 is:

$$
\begin{aligned}
\frac{\partial P_{w,q,r,p}}{\partial t} =& \; k_w(P_{w-1,q,r,p} - P_{w,q,r,p}) + k_q w(P_{w,q-1,r,p} - P_{w,q,r,p}) \\
&+ k_r(q)(P_{w,q,r-1,p} - P_{w,q,r,p}) + k_p r(P_{w,q,r,p-1} - P_{w,q,s,r,p}) \\
&+ g_w\Big[(w+1)P_{w+1,q,r,p} - wP_{w,q,r,p}\Big] + g_q\Big[(q+1)P_{w,q+1,r,p} - qP_{w,q,r,p}\Big] \\
&+ g_r\Big[(r+1)P_{w,q,r+1,p} - rP_{w,q,r,p}\Big] + g_p\Big[(p+1)P_{w,q,s,r,p+1} - pP_{w,q,s,r,p}\Big]. \qquad (12)
\end{aligned}
$$

Introducing the moment generating function as:

$$
F(z_1, z_2, z_3, z_4) = \sum_{w,q,r,p} z_1^w \; z_2^q \; z_3^r \; z_4^p \; P_{w,q,r,p}, \qquad (13)
$$

and using the linearized form of Hill functions in Eq.11, we can convert Eq.12 into a first order partial differential equation (PDE):

$$
\begin{aligned}
\partial_t F =& \; k_w(z_1 F - F) + k_q z_1(z_2 \partial_{z_1} F - \partial_{z_1} F) + k_r^0(z_3 F - F) \\
&+ k_r^1 z_2(z_3 \partial_{z_2} F - \partial_{z_2} F) + k_p z_3(z_4 \partial_{z_3} F - \partial_{z_3} F) \\
&+ g_w(\partial_{z_1} F - z_1 \partial_{z_1} F) + g_q(\partial_{z_2} F - z_2 \partial_{z_2} F) \\
&+ g_r(\partial_{z_3} F - z_3 \partial_{z_3} F) + g_p(\partial_{z_4} F - z_4 \partial_{z_4} F). \qquad (14)
\end{aligned}
$$

This equation cannot be solved exactly but it is not difficult to extract the first two moments of the probability distributions $P_{w,q,r,s}$ at the steady state, thus allowing to obtain a close expression for $p$ and $CV_p = \sigma_p / < p >$.

### 2.2.1 Moments of the distribution

These moments can be evaluated by deriving Eq.14 at the steady state ($\partial_t F = 0$) and using the following properties of the moment generating function: $F|_1 = 1; F_i =< x_i >; F_{ii} =< x_i^2 > - < x_i >$ (with the notation $F_i = \partial_{x_i} F$), where $|_1$ means evaluation of $F$ at $x_i = 1$ for all $i$. We only discuss here the derivatives which are needed to obtain $F_3 =< p >$ and $F_{3,3} - F_3^2 + F_3 = \sigma_p^2$.

$$
\begin{aligned}
F_1 &= k_w/g_w \\
F_2 &= \frac{k_q F_1}{g_q} \\
F_3 &= \frac{k_r^0 + k_r^1 F_2}{g_r} \\
< p >= F_4 &= \frac{k_p F_3}{g_p} \\
F_{1,1} &= \frac{k_w F_1}{g_w} \\
F_{1,2} &= \frac{k_q F_1 + k_w F_2 + k_q F_{1,1}}{g_q + g_w} \\
F_{1,3} &= \frac{k_r^0 F_1 + k_w F_3 + k_r^1 F_{1,2}}{g_r + g_w} \\
F_{1,4} &= \frac{k_w F_4 + k_p F_{1,3}}{g_p + g_w} \\
F_{2,2} &= \frac{k_q F_{1,2}}{g_q} \\
F_{2,3} &= \frac{k_r^0 F_2 + k_r^1 F_2 + k_q F_{1,3} + k_r^1 F_{2,2}}{g_q + g_r} \\
F_{2,4} &= \frac{k_q F_{1,4} + k_p F_{2,3}}{g_p + g_q} \\
\sigma_p^2 - < p > + < p >^2 = F_{3,3} &= \frac{k_r^0 F_3 + k_r^1 F_{2,3}}{g_r} \\
F_{3,4} &= \frac{k_p F_3 + k_r^0 F_4 + k_r^1 F_{2,4} + k_p F_{3,3}}{g_p + g_r} \\
F_{4,4} &= \frac{k_p F_{3,4}}{g_p}.
\end{aligned}
\qquad (15)
$$

## 2.3 The FFL

The master equation describing the circuit in the scheme of Fig. 2 is:

$$
\begin{aligned}
\frac{\partial P_{w,q,s,r,p}}{\partial t} = & \ k_w(P_{w-1,q,s,r,p} - P_{w,q,s,r,p}) + k_q w(P_{w,q-1,s,r,p} - P_{w,q,s,r,p}) \\
& +k_r(q)(P_{w,q,s,r-1,p} - P_{w,q,s,r,p}) + k_s(q)(P_{w,q,s-1,r,p} - P_{w,q,s,r,p}) \\
& +k_p(s)r(P_{w,q,s,r,p-1} - P_{w,q,s,r,p}) + g_w\Big[(w+1)P_{w+1,q,s,r,p} - wP_{w,q,s,r,p}\Big] \\
& +g_q\Big[(q+1)P_{w,q+1,s,r,p} - qP_{w,q,s,r,p}\Big] + g_r\Big[(r+1)P_{w,q,s,r+1,p} - rP_{w,q,s,r,p}\Big] \\
& +g_s\Big[(s+1)P_{w,q,s+1,r,p} - sP_{w,q,s,r,p}\Big] + g_p\Big[(p+1)P_{w,q,s,r,p+1} - pP_{w,q,s,r,p}\Big]. \qquad (16)
\end{aligned}
$$

Introducing the moment generating function:

$$
F(z_1, z_2, z_3, z_4, z_5) = \sum_{w,q,s,r,p} z_1^w \ z_2^q \ z_3^s \ z_4^r \ z_5^p \ P_{w,q,s,r,p}, \qquad (17)
$$

and using the linearization in Eqs.9, we can convert Eq.26 into a PDE that is of second order in this case:

$$
\begin{aligned}
\partial_t F = & \ k_w(z_1 F - F) + k_q z_1(z_2 \partial_{z_1} F - \partial_{z_1} F) + k_r^0(z_4 F - F) \\
& +k_r^1 z_2(z_4 \partial_{z_2} F - \partial_{z_2} F) + k_s^0(z_3 F - F) + k_s^1 z_2(z_3 \partial_{z_2} F - \partial_{z_2} F) \\
& +k_p^0 z_4(z_5 \partial_{z_4} F - \partial_{z_4} F) - k_p^1 z_3 z_4(z_5 \partial_{z_3, z_4} F - \partial_{z_3, z_4} F) \\
& +g_w(\partial_{z_1} F - z_1 \partial_{z_1} F) + g_q(\partial_{z_2} F - z_2 \partial_{z_2} F) + g_s(\partial_{z_3} F - z_3 \partial_{z_3} F) \\
& +g_r(\partial_{z_4} F - z_4 \partial_{z_4} F) + g_p(\partial_{z_5} F - z_5 \partial_{z_5} F). \qquad (18)
\end{aligned}
$$

As mentioned in the main text, even if we linearized the Hill functions in the master equation (Eq.26), the term related to the translation of the regulated target keeps a nonlinear contribution due to the product $k_p^1 sr$. This has the effect of making Eq.18 a second order PDE.
Remarkably enough one can nevertheless obtain closed analytical expressions for $< p >$ and $CV_p$. The only additional complication, with respect to TF-gene case discussed in the previous section is that the calculation of some fourth moments is required. We do not report here the expression of all the moments for the sake of shortness but they can be easily derived with tedious but straightforward algebra.

## 2.4 The open circuit

The master equation describing the circuit in the scheme of Fig. 3 is:

$$\frac{\partial P_{w,q,w',q',s,r,p}}{\partial t} = k_w \Big[(P_{w-1,q,w',q',s,r,p} - P_{w,q,w',q',s,r,p})$$
$$+(P_{w,q,w'-1,q',s,r,p} - P_{w,q,w',q',s,r,p})\Big]$$
$$+k_q \Big[w(P_{w,q-1,w',q',s,r,p} - P_{w,q,w',q',s,r,p})$$
$$+w'(P_{w,q,w',q'-1,s,r,p} - P_{w,q,w',q',s,r,p})\Big]$$
$$+k_r(q)(P_{w,q,w',q',s,r-1,p} - P_{w,q,w',q',s,r,p})$$
$$+k_p(s)r(P_{w,q,w',q',s,r,p-1} - P_{w,q,w',q',s,r,p})$$
$$+k_s(q')(P_{w,q,w',q',s-1,r,p} - P_{w,q,w',q',s,r,p})$$
$$+g_s\Big[(s+1)P_{w,q,w',q',s+1,r,p} - sP_{w,q,w',q',s,r,p}\Big]$$
$$+g_w\Big[(w+1)P_{w+1,q,w',q',s,r,p} - wP_{w,q,w',q',s,r,p}\Big]$$
$$+g_q\Big[(q+1)P_{w,q+1,w',q',s,r,p} - qP_{w,q,w',q',s,r,p}\Big]$$
$$+g_w\Big[(w'+1)P_{w,q,w'+1,q',s,r,p} - w'P_{w,q,w',q',s,r,p}\Big]$$
$$+g_q\Big[(q'+1)P_{w,q,w',q'+1,s,r,p} - q'P_{w,q,w',q',s,r,p}\Big]$$
$$+g_r\Big[(r+1)P_{w,q,w',q',s,r+1,p} - rP_{w,q,w',q',s,r,p}\Big]$$
$$+g_p\Big[(p+1)P_{w,q,w',q',s,r,p+1} - pP_{w,q,w',q',s,r,p}\Big]. \quad (19)$$

Introducing the moment generating function:

$$F(z_1,z_2,z_3,z_4,z_5,z_6,z_7) = \sum_{w,q,w',q',s,r,p} z_1^w\, z_2^q\, z_3^{w'}\, z_4^{q'}\, z_5^s\, z_6^r\, z_7^p\, P_{w,q,w',q',s,r,p}, \quad (20)$$

and using the linearization in Eqs.9 we can convert Eq.19 into a second order PDE analogous to Eq.18. The expression of $< p >$ and $CV_p$ can be obtained as in the FFL case differentiating up to fourth moments.

## 2.5 Numerical definition of the steady-state time-threshold

To perform Gillespie simulations in order to check the analytical results, we must define the time $t_c$ at which the system can be considered at the steady state. In previous papers [1, 2] the steady state was assumed to be reached at a time equal to ten times the protein half-life. We tried to slightly improve this definition.

For each circuit in analysis, we evaluated numerically the deterministic dynamics for the set of parameter values chosen for the simulations, assuming the initial conditions $x_i = 0\ \forall i$. Then we defined $t_c$ as the value above which the difference between $p(t_c)$ and its asymptotic value $p_{ss}$ (in units of $p_{ss}$) becomes smaller than a given threshold $\epsilon$.

$$\frac{p_{ss} - p(t_c)}{p_{ss}} = \epsilon. \quad (21)$$

We report in fig.4 an example of this calculation in the case of the FFL circuit (details on the parameters and initial conditions are reported in the caption). Setting as threshold $\epsilon = 0.05$ (which,

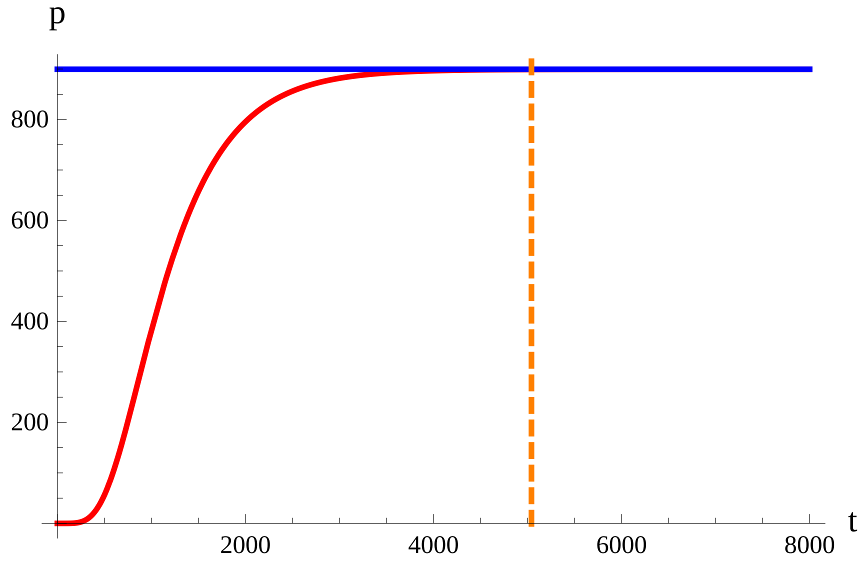


**Figure 4.** Deterministic evolution toward the steady state. We report in blue the numerical solution of Eqs.4 for $p$ with initial conditions $x_i = 0\ \forall i$. The red line represents the number of protein at equilibrium given by Eq.6. The dashed orange line represents the time $t_c$ that satisfies Eq.21. At time $t_c = 5000s$, resulting from Eq.21 with $\epsilon = 0.05$, the curves are almost indistinguishable. The rate of transcription of the TF is $k_w = 0.06s^{-1}$ and of translation $k_q = 0.04s^{-1}$. Proteins degrade with a probability $g_q = g_p = 0.002s^{-1}$ while mRNAs and miRNAs with probability $g_w = g_r = g_s = 0.006s^{-1}$. The parameters in the Hill functions (Eqs.5) are as follows. Maximum rate of transcription for mRNAs: $k_r = 0.8s^{-1}$, while for miRNAs: $k_s = 0.5s^{-1}$; maximum rate of translation of the target: $k_p = 0.04s^{-1}$; dissociations constants: $h_s = 200, h_r = 200, h = 60$. The Hill coefficients are always $c = 2$.

given the size of the fluctuations which we are interested in, turns out to be a rather conservative value) we found for this particular circuit $t_c \sim 5000s$, which corresponds to about 14 times the protein half-life.

This procedure allows us to treat the different circuits on the same ground and eliminates a possible source of numerical bias.

## 2.6 Robustness of our results with respect to changes in the values of the input parameters

### 2.6.1 Constraints imposed on the FFLs by the requirement of sensitivity to changes in the master TF concentration.

Functional FFLs can be defined as those in which a change in the master TF concentration can cause a change in the concentration of target proteins and miRNAs [3]. While the issue of the trade-off between sensitivity to signals and noise control is discussed in detail in Section 7, in the following we shall define more simply the conditions on parameters that ensure a sufficient dependence of miRNA and target mRNA levels on the TF concentration. Noise propagation requires a target dependence on TF concentration, therefore only in this case noise buffering can be functional. In our contest this dependence implies that the Hill functions of activation by the TF and of repression by the miRNA should not be saturated at the steady state. Indeed, in conditions of complete saturation, signals and fluctuations cannot propagate from the master TF to the target (even in absence of miRNA regulation), therefore a noise control lose any functionality. On the other hand, in the unsaturated regime a change in the number

of TFs can alter in a significant way the number of target proteins in the cell together with the number of miRNAs, generating the correlated fluctuations needed for noise buffering. If the TF concentration is too high (with respect to $h_r$ and $h_s$), the expression rates of the target and miRNA genes become insensitive to variations in TF concentration (unless they are so large that can escape from the region of saturation) limiting the sensitivity of the FFL to upstream signals. The same considerations hold for the target repression. If there are too many miRNAs (with respect to $h$), the target expression is drastically shut down and again the system becomes insensitive to changes in the number of TFs. Accordingly we excluded from our analysis the parameter sets for which: $< q > \ll h_r(h_s)$ or $< q > \gg h_r(h_s)$ and $< s > \ll h$ or $< s > \gg h$. In other words, the circuit functionality imposes that concentrations of regulators must be placed not far from the linear region of the corresponding Hill functions. A high sensitivity corresponds also to an overexposure to noise, in fact noise amplification and sensitivity are correlated quantities [4,12] (see also Section 7). Since the aim of our study is to prove the noise buffering role of miRNA-mediated incoherent FFL, considering the parameter space that strongly exposes to noise makes clearly sense and it seems not a limitation.
With the conditon of unsaturated regulations satisfied, the qualitative results in the article apply for virtually all parameter choices. As a partial proof, in the next two sections we shall discuss a few different combinations of parameters. As we shall see our results turn out to be remarkably robust with respect to changes in the allowed (unsaturated) region of parameters.

#### 2.6.2 Target and miRNA genes differentially expressed

In this section we present the target noise strength for the three circuits as a function of the ratio between the maximum rate of transcription of miRNA gene ($k_s$) and target gene ($k_r$), keeping fixed the TF concentration ($< q >$) and miRNA repression strength ($1/h$). The aim is to show that the noise buffering role of the mixed FFL shows only a weak dependence on the characteristics of miRNA and target promoters. In the upper part of Fig.5 we plot the target noise strength as a function of $k_r/k_s$. One can see that in the whole range of values the mixed FFL shows the largest noise reduction effect and in particular that the noise buffering role of the FFL does not require an equal rate of transcription of miRNAs and mRNAs. Indeed, as discussed in the main text, the noise attenuation is due to the correlation of fluctuations in the number of mRNAs and miRNAs and not to their absolute values. Different maximum rates of transcription ($k_r$ and $k_s$) only change the height of peaks in mRNA and miRNA trajectories, without affecting their correlation.

#### 2.6.3 mRNAs and miRNAs with different stability

Another important robustness test is the dependence of the FFL noise buffering efficiency on the ratio of decay constants $g_r/g_s$. In principle one could expect a reduction in the FFL efficiency when $g_r \neq g_s$ due to the fact that with different values of $g_r$ and $g_s$ the mRNA and miRNA trajectories could start to fluctuate out of phase due to different relaxation times. To answer this question we calculated the $CV_p$ for the three circuits as a function of the ratio $g_r/g_s$. The results are reported in the lower part of Fig.5.

As in the previous case, we find that in the whole range of $g_r/g_s$ that we studied the mixed FFL gives the largest noise reduction effect.

These two tests together show that noise buffering is a generic feature of mixed FFLs and that there is no need to fine tune the half-life and/or the transcription rate of miRNAs and mRNAs to obtain a mixed FFL that efficiently reduces fluctuations.

#### 2.6.4 Optimal TF concentration tuning $k_w$ instead of $k_q$

In the main text we discussed the dependence of the noise strength $CV_p$ on the copy number of TFs present at the steady state (Fig.6C of the main text). The parameter chosen to tune $< q >$ was the

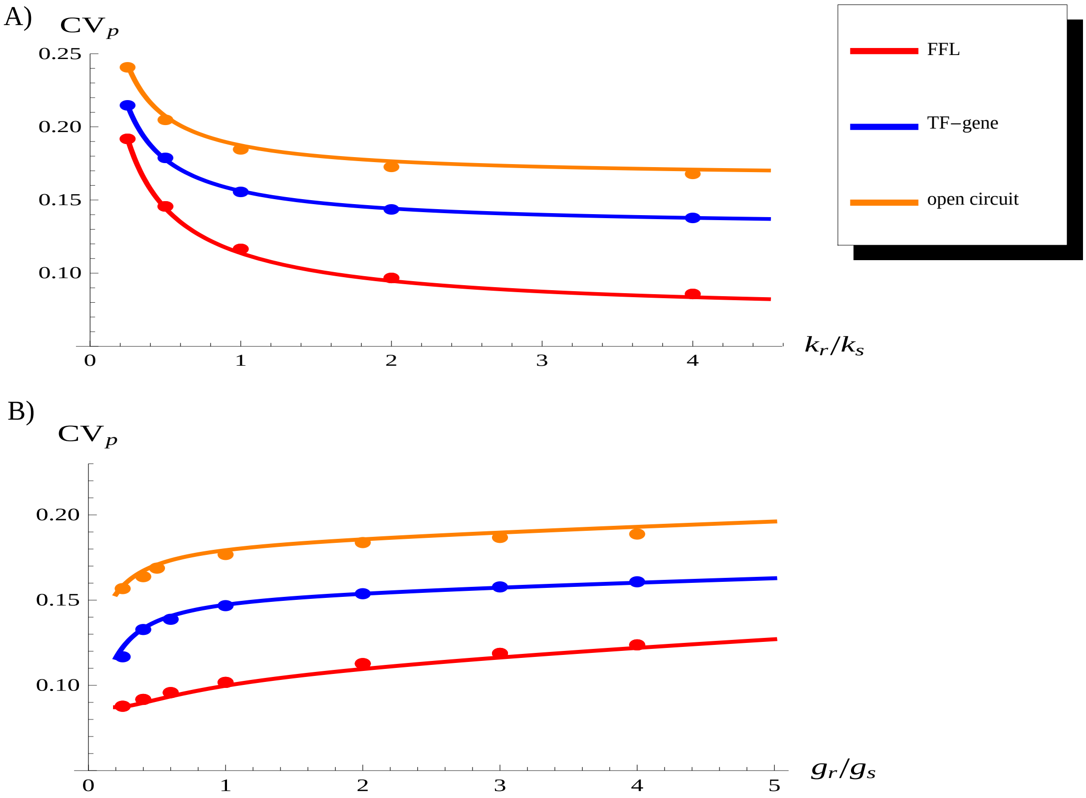


**Figure 5.** (A) $CV_p$ as a function of the transcription rate ratio $k_r/k_s$. (B) $CV_p$ as a function of $g_r/g_s$.

rate of translation $k_q$. For the sake of completeness we report here the same plot obtained by varying $k_w$ instead of $k_q$. Also with this alternative protocol the FFL outperforms the other circuits in noise control for intermediate concentration of the TF. This is a further proof of the robustness of our results.

### 2.6.5 Results for another set of parameters

As a final test of robustness we solved the master equations for the three circuits with a choice of input parameters (reported in the caption of Fig.7A) leading to sizeable fluctuations in the number of master TFs ($CV_q \sim 0.4$). This should be compared with the values of the case dicussed in the main text (whose parameter set is reported in the caption of Fig.4) for which the noise in the number of TFs was only $CV_q \sim 0.17$. Also in this case the TF fluctuations are efficiently attenuated by the FFL, leading to a final value of the noise strength in the target protein of $CV_p = 0.25$ to be compared with $CV_p = 0.38$ for the direct TF-gene regulation and $CV_p = 0.46$ for the open circuit (see the histograms in Fig.7A). These values agree with the observation reported in the main text that the noise attenuation effect due to the FFL circuit becomes larger and larger as the size of TF fluctuations increases. The U-shaped profile of $CV_p$ for the FFL steps out also for this parameter set, further supporting the idea that this property does not depend on their particular choice but is a generic feature of the model (see Fig.7B).

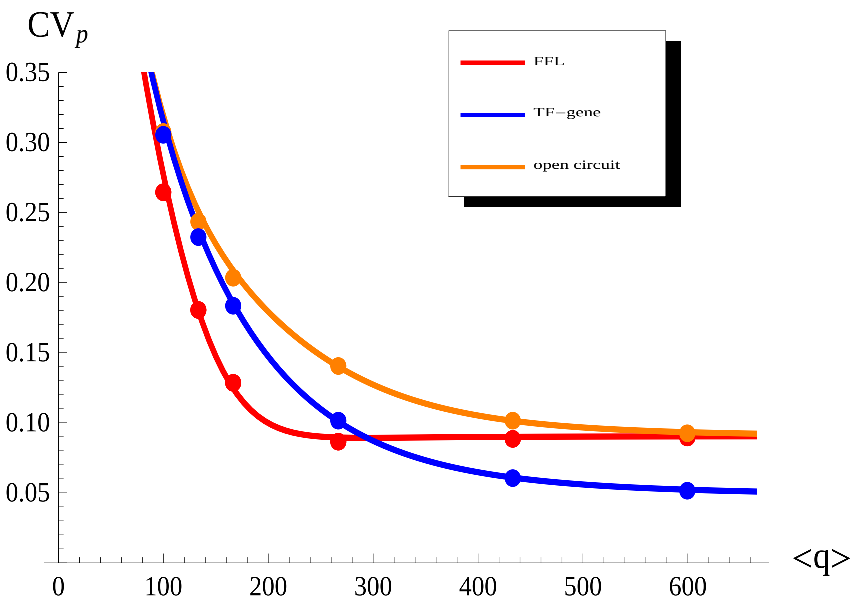


**Figure 6.** $CV_p$ as a function of the mean number of TFs $< q >$. Dots are the result of Gillespie simulations with the full dynamics while continuous lines are the analytical predictions. The parameter values are the same of Fig.4

### 2.7 Testing the effect of Hill function linearization

Besides the robustness against the choice of input parameters another important issue which one would like to address is the effect of the linearization of the Hill functions. This can be easily achieved by comparing analytical versus numerical (Gillespie) results for the noise reduction. Since this is the only approximation that we made in our analysis it is important to understand which is the range of parameters in which we can trust our analytical results not only qualitatively but also quantitatively. It is easy to guess that the linear approximation should give sizeable errors only when the fluctuations in the variables become large enough to cover a wide portion of the Hill function thus exploring also its non-linear part. A good example to discuss this issue is given by the set of input parameters discussed in the previous section. In this case, even if the analytical solution still captures qualitatively the main features of the systems, it is less precise in its quantitative predictions. This is clearly visible in Fig.7B where analytical predictions are compared with the results of Gillespie simulations (which keep into account the full non linear dynamics of the FFL) as a function of the inverse of repression strength $h$. While for the value of $h$ discussed above ($h = 30$) the agreement is very good, as $h$ decreases the gap between the two curves becomes larger and larger. This is a consequence of the linearization of Hill functions and shows that if fluctuations are too large, as it happens in the strong repression regime, the linear approximation may become too crude. It is interesting to study how the approximation breaks down since it is a typical example of the subtle effects which the two step nature of gene expression may have on noise propagation. With the choice of parameters of the figure, the $q$ fluctuations cover a wide region of the domain of $k_r(q)$ and $k_s(q)$ (Fig.7C), but the line tangent in $< q >$ still captures quite well the Hill function trend, with only a slight overestimation ($< r >=< s >= 20$ from simulations, compared to the predicted value of 21). On the other hand, the large fluctuations in $s$ ($CV_s = 0.48$) make the linearization of $k_p(s)$ a poor approximation (Fig.7D). The $s$ distribution spreads on a domain region where the Hill function widely changes its curvature, therefore the tangent line introduces in many trajectories a sizeable

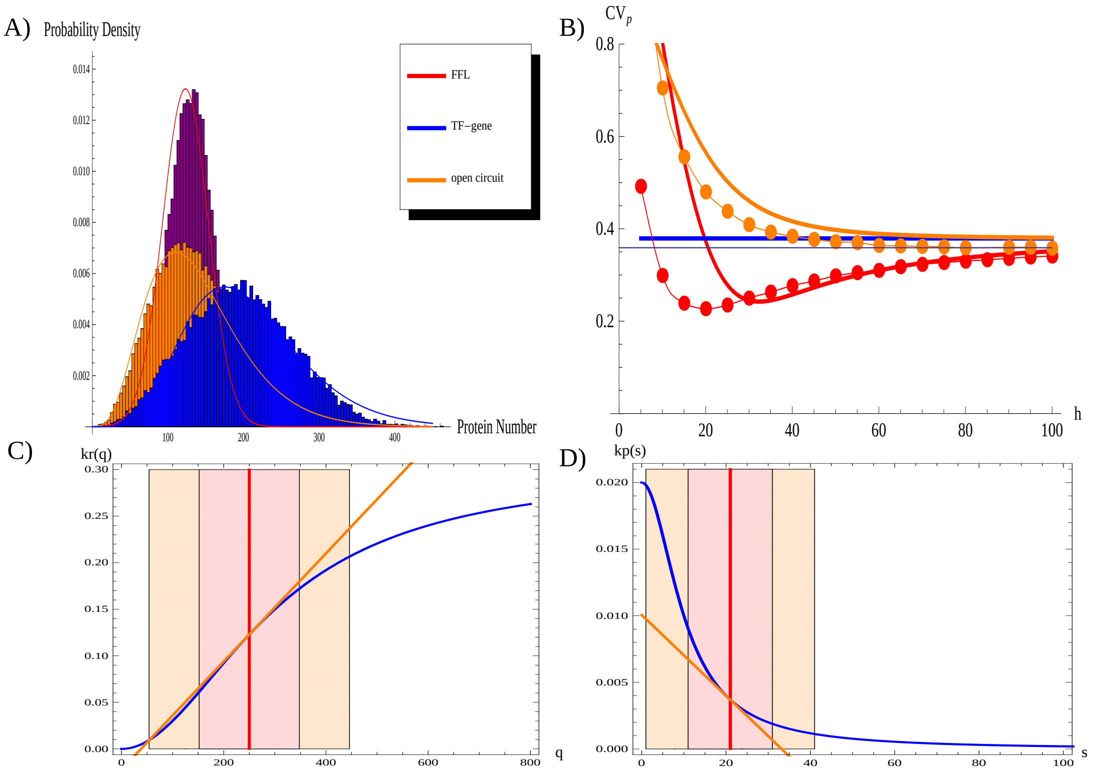


**Figure 7.** (A)The probability distribution of target protein number for the three circuits in analysis. The parameter values are: $k_w = 0.01s^{-1}$; $k_q = 0.3s^{-1}$; $g_w = g_r = g_s = 0.006s^{-1}$; $g_q = g_p = 0.002s^{-1}$; $k_r = k_s = 0.3s^{-1}$; ; $h_s = h_r = 200$; $c = 2$; $h = 30$. Histograms are the result of Gillespie simulations with the full nonlinear dynamics, while continuous lines are empirical distributions (gaussian for the FFL and gamma for the TF-gene and the open circuit) with mean and variance predicted by the analytical model. (B) The coefficient of variation of the target protein $CV_p$ as a function of the inverse of repression strength $h$ for the three circuits. (C) The Hill function of transcriptional activation of the target gene (blue line). The red line represents the mean number of TFs $< q >$ at equilibrium, while the shaded region corresponds to intervals $[q - \sigma_q, q + \sigma_q]$ and $[q - 2\sigma_q, q + 2\sigma_q]$. The orange line represents the linearized function used for the analytical solution. (D) The Hill function of translational repression of the target gene (blue line) by the miRNA, in the strong repression region ($h = 10$). The red line represents the mean number of miRNAs $< s >$ at equilibrium, while the shaded region corresponds to intervals $[s - \sigma_s, s + \sigma_s]$ and $[s - 2\sigma_s, s + 2\sigma_s]$. The orange line represents the linearized function used for the analytical solution.

underestimation of the rate of target translation. As a result we have $< p >= 43$ from simulations while only $< p >= 28$ from the analytical model. In a similar way also the standard deviation turns out to be uncorrectly estimated by the analytical solution. These disagreements explain the displacement of analytical curves in Fig.7B with respect to simulations. This example shows however that, despite its quantitative failure, the analytical model describes fairly well the qualitative behaviour of the system even in presence of large fluctuations and, as mentioned above, it becomes more and more precise when fluctuations around steady state values cover a domain where the Hill functions are approximately linear (which is the usual assumption in literature).

# 3 MiRNA-mediated promotion of mRNA degradation

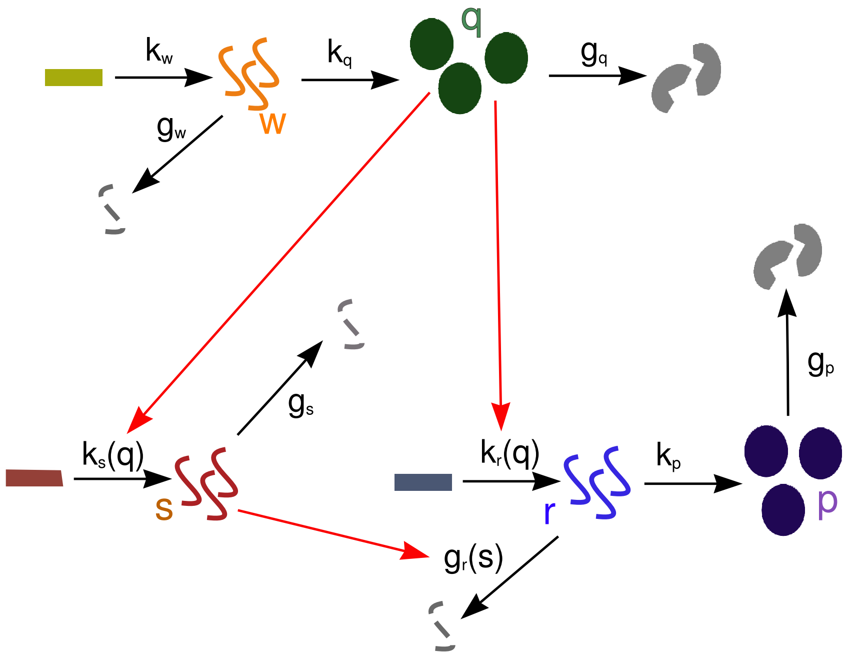


**Figure 8.** Scheme of a miRNA-mediated incoherent FFL, where the miRNA performs its repressive function by promoting mRNA degradation. The notation is the same of Fig.1. The red arrow starting from $s$ represents the regulation of the rate of degradation $g_r(s)$, which in this case is a non-linear increasing function of miRNA concentration.

By base pairing to mRNAs, miRNAs can mediate translational repression or mRNA degradation [5–7]. As discussed in the main text, we developed our model considering the miRNA action as repressing the target translation, making the rate of mRNA translation a nonlinear decreasing function of the number of miRNAs. In this section we will prove the validity of our results even in the case of miRNA repression based on promotion of target mRNA degradation. In this case we can introduce the miRNA action adding to the basal rate of mRNA degradation $g_r$ (in absence of miRNAs) an increasing Hill function of the copy number of miRNAs:

$$g_r(s) = g_r + \frac{g_{max} s^c}{h_{deg}^c + s^c}, \tag{22}$$

where $g_{max}$ represents the maximum possible increase of the degradation rate in case of high miRNA concentration (if $s \to \infty, g_r(s) \to g_r + g_{max}$); $h_{deg}$ is the dissociation constant of miRNA-mRNA interaction; $c$ is the Hill coefficient.

The stochastic models built on this assumptions cannot be solved with the same strategy explained in section 2. The closure of equations for $< p >$ and $\sigma_p$ would require further linearizations. However, we ran simulations for the alternative mechanism of miRNA-mediated promotion of target mRNA degradation to check the robustness of our results. Strikingly enough these simulations can be fit quite well with the analytical predictions based on the assumption of a miRNA-mediated repression of translation.

## 3.1 Deterministic model

The TF-gene linear circuit is modelled as previously shown. We present here only the deterministic equations for the FFL, since the open circuit case can be easily obtained from the FFL description following the same steps discussed above for the translational repression case. The mean field description of the system in Fig.8 is:

$$\begin{aligned} \frac{dw}{dt} &= k_w - g_w w \\ \frac{dq}{dt} &= k_q w - g_q q \\ \frac{ds}{dt} &= k_s(q) - g_s s \\ \frac{dr}{dt} &= k_r(q) - g_r(s) r \\ \frac{dp}{dt} &= k_p r - g_p p, \end{aligned} \tag{23}$$

where $k_s(q)$ and $k_r(q)$ are the Hill functions of activation shown in Eqs.5, while the form of $g_r(s)$ is shown in Eq.22.
Assuming $c = 2$ the expressions at steady state of $w_{ss}, q_{ss}, s_{ss}$ are the same of Eqs.6, as nothing is changed in their dynamics, while the expressions of $r_{ss}$ and $p_{ss}$ become:

$$\begin{aligned} r_{ss} &= \frac{k_q^2 k_r k_w^2 (g_q^4 g_s^2 g_w^4 h_{deg}^2 h_s^4 + 2 g_q^2 g_s^2 g_w^2 h_{deg}^2 h_s^2 k_q^2 k_w^2}{(g_q^2 g_w^2 h_r^2 + k_q^2 k_w^2)(g_q^4 g_r g_s^2 g_w^4 h_{deg}^2 h_s^4 + 2 g_q^2 g_r g_s^2 g_w^2 h_{deg}^2 h_s^2 k_q^2 k_w^2} \cdot\cdot \\ &\quad \frac{+k_q^4 (g_s^2 h_{deg}^2 + k_s^2) k_w^4)}{+k_q^4 (g_r g_s^2 h_{deg}^2 + (g_r + g_{max}) k_s^2) k_w^4)} \\ p_{ss} &= r_{ss} k_p / g_p. \end{aligned} \tag{24}$$

## 3.2 Comparison with miRNA-mediated repression of mRNA translation

In order to compare in an unbiased way the noise properties of the mixed FFL with different mechanisms of miRNA action, we set up the parameters of the two alternative models (Fig.2 and 8) so as to achieve the same final levels of the target protein $p_{ss}$. This can be obtained by choosing the same parameters for the two models except those involved in the miRNA regulation. These last may then be fixed by equating the values of $p_{ss}$ in Eq.6 and 24. As show in Fig.9 the result of this comparison is that a mixed FFL with a degradation-based repression gives essentially the same results of the corresponding circuit with a translation-based repression.

In particular, we show in Fig.9A the analogous (for the present repression scheme) of the histograms of Fig.3C and 4C of the main text. As in the translational repression case also in this model the noise buffering effect of the FFL is clearly visible thus suggesting that the inchoerent FFL loop performs equally well its noise buffering function with either type of repression mechanism. Superimposing the distributions with mean and variance calculated analytically for the miRNA-mediated repression of translation we find again a very good agreement, apart from a slight disagreement in the strong repression regime (small $h$). In conclusion, all the results presented in the main paper hold despite the mechanism of miRNA repression and even if the analytical predictions are based on the assumption of a miRNA-mediated repression on mRNA translation, they can be applied also to this case.

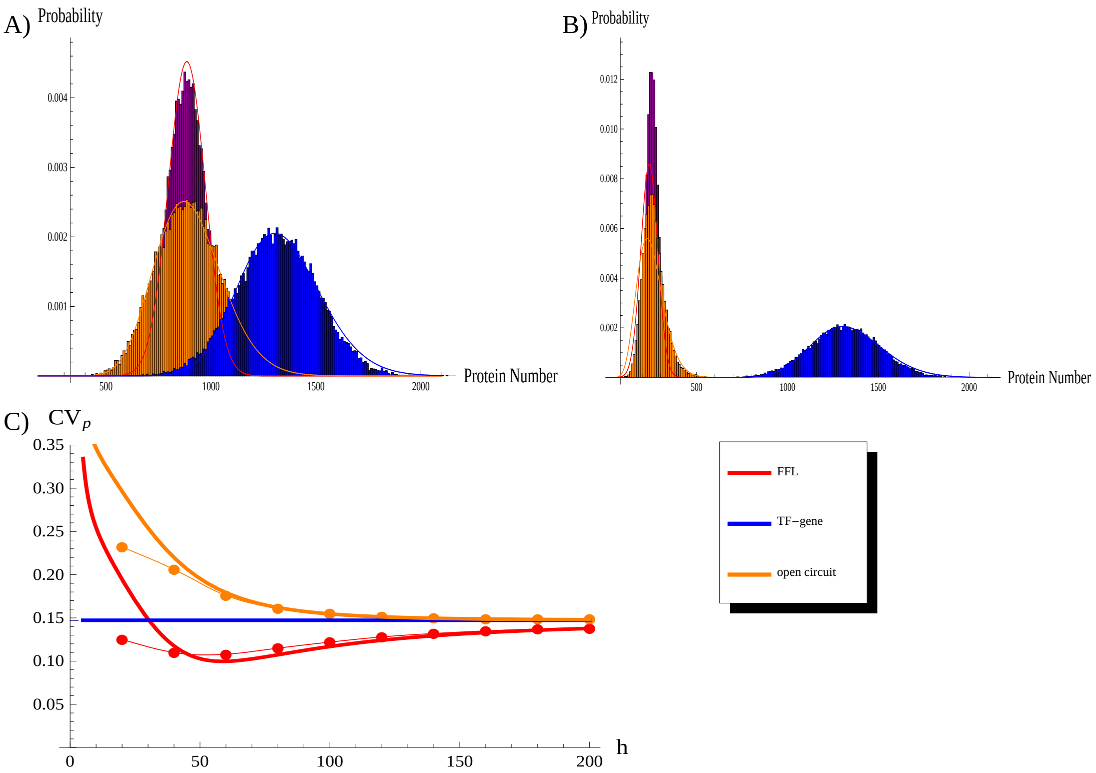


**Figure 9.** Attenuation of noise by a FFL with a miRNA promoting degradation. (A) The probability distribution of target protein number for the three circuits in analysis. Histograms are the result of Gillespie simulations with the nonlinear dynamics depicted in Fig.8. Continuous lines are empirical distributions (gaussian for the FFL and gamma for the TF-gene and the open circuit) with mean and variance predicted by the analytical stochastic model shown in section 2.3. The parameter values are those explained in caption of Fig.4. Even if the analytical model is built on the hypothesis of repression of mRNA-translations, it fits equally well the distributions resulting from simulations based on miRNA-mediated promotion of mRNA degradation. (B) Same histograms of A with a stronger repression ($h = 20$, all other parameters as stated before). In the regime of strong repression the analytical model tends to overestimate the variance $\sigma_p$. (C) The coefficient of variation of the target protein $CV_p$ as a function of the inverse of repression strength $h$ for the three circuits. The figure shows the presence of an optimal repression strength even in the case of a degradation-based miRNA repression. Dots are the results of Gillespie simulations with the hypothesis of a miRNA-mediated promotion of mRNA degradation, while thick lines are analytical predictions. Apart from the mentioned overestimation in the strong repression region the model fits quite well Gillespie simulations.

# 4 Stoichiometric mechanism of repression

Regulatory small noncoding RNAs (sRNAs) play a crucial role also in prokaryotes gene regulation. In particular, the class of *trans*-acting sRNAs has many features in common with miRNAs in eukaryotes: most of them bind to the UTR of the target mRNAs through base-pairing (often imperfect) recognition to prevent their translation or to promote their degradation. However, as discussed in [8], unlike their eukaryotic counterpart they usually act stoichiometrically on their targets, since a given sRNA molecule is often degraded along with its target, instead of being used to regulate other targets. Different au-

thors [8–10] studied the peculiar features of this noncatalytic sRNA-mediated regulation, developing a simple kinetic model for sRNA gene silencing.
In this section we shall study the noise buffering properties of incoherent FFL motifs assuming a stoichiometric modality of repression, and compare our results with the previously discussed catalytic case.

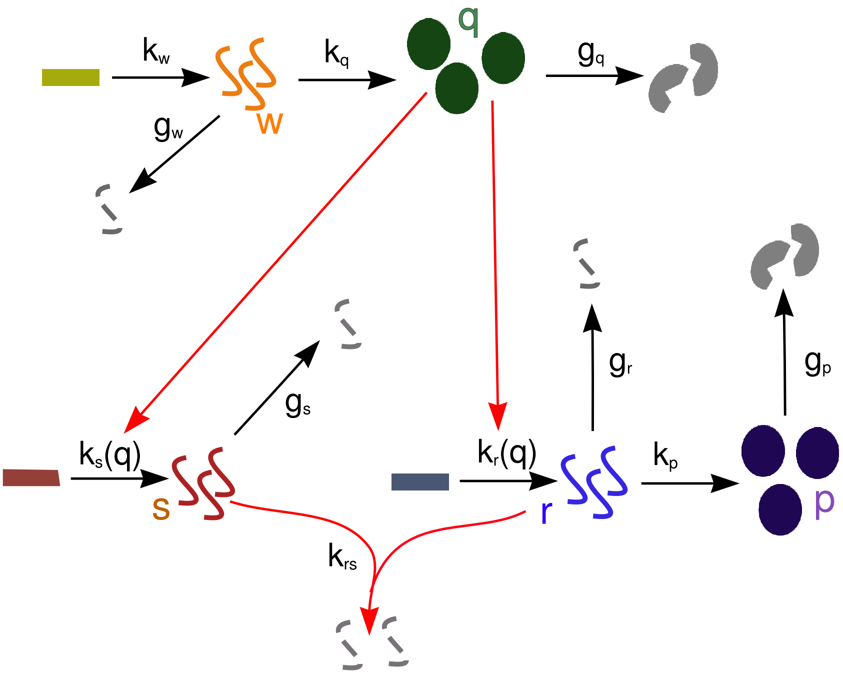


**Figure 10.** Scheme of a miRNA-mediated incoherent FFL, where pairing of miRNA and mRNA exposes both molecules to co-degradation. The coupled degradation of the miRNA-mRNA pair is described through a second-order kinetic constant $k_{rs}$ .

## 4.1 Deterministic model

The scheme of a mixed FFL in which the coupling between sRNAs ($s$) and mRNAs ($r$) is stoichiometric is depicted in Fig.10. Following [8–10], we assume that both sRNA and mRNA are co-degraded when paired with a rate that depends on the sRNA-mRNA interaction strength $k_{rs}$. The mean field kinetic of our system can be described by the equations:

$$\begin{aligned}
\frac{dw}{dt} &= k_w - g_w w \\
\frac{dq}{dt} &= k_q w - g_q q \\
\frac{ds}{dt} &= k_s(q) - g_s s - k_{rs} r s \\
\frac{dr}{dt} &= k_r(q) - g_r r - k_{rs} r s \\
\frac{dp}{dt} &= k_p r - g_p p.
\end{aligned} \qquad (25)$$

The stationary solutions ($d_t x_i = 0 \ \forall i \in \{w, q, s, r, p\}$) can be easily calculated (not reported).

### 4.2 Comparison with catalytic repression

In this section we will explore the consequences of the nature of sRNA-mRNA interaction (stoichiometric or catalytic) on the noise properties of the mixed FFL. In analogy to section 3.2, we shall compare the two models choosing the parameters so as to obtain the same $p_{ss}$ with both types of sRNA action. As can be seen comparing the schemes in Fig.3 and Fig.10, in order to have the same number of target proteins at equilibrium we set equal rates of production and degradation of each molecular species and then find the relation between $k_{rs}$ in the stoichiometric model and $h$ in the catalytic model by equating the expression for $p_{ss}$ in Eqs.6 and in the solution of Eqs.25.

We ran simulations for the FFL, TF-gene direct regulation and the open circuit for catalytic and stoichiometric action (Fig.11). The noise filtering effect is robust with respect to the mechanism of miRNA-mRNA interaction, but a catalytic interaction makes the FFL more efficient in buffering fluctuations (compare the histograms in Fig.11A and B). The U-shaped profile of the noise strength $CV_p$ of a target controlled by a FFL (discussed in the main text for catalytic repression) is recovered also in the stoichiometric case. We report in Fig.11C and D the $CV_p$ as a function of the inverse of repression strength for two different sets of parameter values. The maximum of attenuation is achieved for approximately the same value of $h$ of the catalytic case but the size of noise reduction is smaller with a stoichiometric repression.

As reported in [9] and [11], in the stoichiometric model described by Eqs.25 the mean protein number exhibits a threshold linear behaviour as a function of the ratio $k_r/k_s$ with the threshold in 1 [9]. Following [8], protein expression can be classified into three regimes: repressed ($k_r/k_s \ll 1$), crossover ($k_r/k_s \approx 1$) and expressing ($k_r/k_s \gg 1$). A threshold linear behaviour implies ultrasensitivity in the crossover regime and as a consequence the noise is enhanced near the threshold due to critical fluctuations ( [8] and references therein). However, this threshold-linear response is expected if the mRNA-sRNA interaction is strong, while for a weaker repression the threshold smoothly disappears (see Fig.12) and the three regimes become indistinguishable. The analysis presented in Fig.11 shows that the attenuation of fluctuations by a mixed FFL is observable in a regime of weak repression, corresponding to $k_{rs} \sim 10^{-4}$ in Fig.12, where the crossover regime is vanishing and the raise in fluctuation in $k_r/k_s = 1$ is negligible.

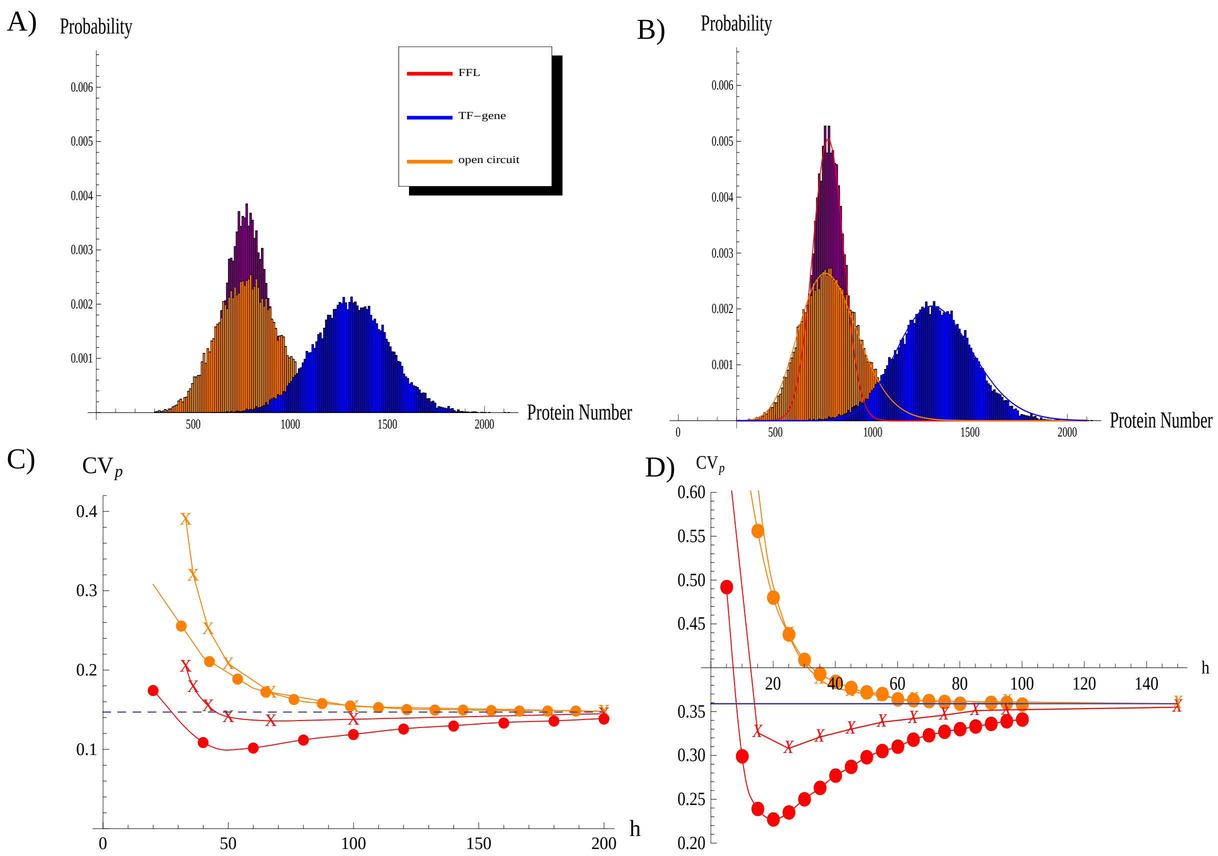


**Figure 11.** Attenuation of noise by a FFL mediated by a sRNA that acts stoichiometrically on its mRNA target. We chose the same parameter set described in the caption of Fig.4. In the upper part of the figure we show the probability distribution of the target protein number for the three circuits in the case of stoichiometric action (A) and catalytic action (B). Although in both cases the FFL reduces relative fluctuations with respect to the direct TF regulation and the open circuit, the catalytic modality turns out to be more efficient than the stoichiometric one. For the same set of parameters we report in (C) the $CV_p$ as a function of the inverse of the repression strength $h$. In the stoichiometric case $CV_p$ is actually a function of $k_{rs}$ which however can be expressed as function of $h$ (see the text). To allow a simpler comparison of the various plots we plotted the stoichiometric results directly as function of $h$. Dots are the result of simulations based on the hypothesis of a catalytic sRNA action while the x-shaped points derive from simulations with a stoichiometric action. For each regulatory modality we report the FFL and the open circuit data (which can be recognized because are always higher than the FFL ones). Even if the qualitative behaviour is the same (in both cases a maximum of noise attenuation appears) the figure clearly shows that the catalytic modality is more efficient than the stoichiometric one in reducing the noise. (D) Same as (C) but for the alternative set of parameters discussed in the caption of Fig.7.

# 5 Purely transcriptional incoherent FFLs

## 5.1 Stochastic model

The master equation describing a purely transcriptional incoherent FFL (depicted in the scheme of Fig. 13) is:

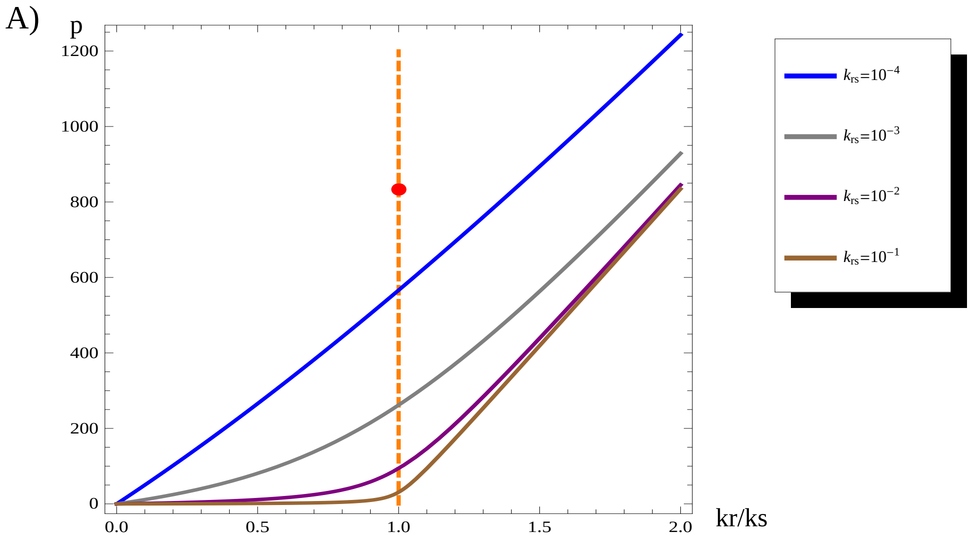


**Figure 12.** We report the response ($p$) of the FFL as a function of $k_r/k_s$ for different values of miRNA-mRNA interaction strength. The red dot represents the protein production in absence of miRNA regulation. The threshold linear response is evident only for strong repression, while for $k_{rs} = 10^{-4}$, compatible with a fine tuning regulation, the response is almost purely linear.

$$
\begin{aligned}
\frac{\partial P_{w,q,s,j,r,p}}{\partial t} = \quad & k_w(P_{w-1,q,s,j,r,p} - P_{w,q,s,j,r,p}) + k_q w(P_{w,q-1,s,j,r,p} - P_{w,q,s,j,r,p}) \\
& + k_s(q)(P_{w,q,s-1,j,r,p} - P_{w,q,s,j,r,p}) + k_j s(P_{w,q,s,j-1,r,p} - P_{w,q,s,j,r,p}) \\
& + k_r(q,j)(P_{w,q,s,j,r-1,p} - P_{w,q,s,j,r,p}) + k_p r(P_{w,q,s,j,r,p-1} - P_{w,q,s,j,r,p}) \\
& + g_w\Big[(w+1)P_{w+1,q,s,j,r,p} - wP_{w,q,s,j,r,p}\Big] + g_q\Big[(q+1)P_{w,q+1,s,j,r,p} - qP_{w,q,s,j,r,p}\Big] \\
& + g_s\Big[(s+1)P_{w,q,s+1,j,r,p} - sP_{w,q,s,j,r,p}\Big] + g_j\Big[(j+1)P_{w,q,s,j+1,r,p} - jP_{w,q,s,j,r,p}\Big] \\
& + g_r\Big[(r+1)P_{w,q,s,j,r+1,p} - rP_{w,q,s,j,r,p}\Big] + g_p\Big[(p+1)P_{w,q,s,j,r,p+1} - pP_{w,q,s,j,r,p}\Big]. \qquad (26)
\end{aligned}
$$

The protein $j$ represses target transcription, which is also activated by the master TF $q$; consequently the rate $k_r(q,j)$ is represented as a function of the concentration of both regulators. In particular, we model the rate of target transcription as a product of Hill functions:

$$
k_r(q,j) = k_r \frac{q^c}{h_r^c + q^c} \frac{1}{1 + (\frac{j}{h_j})^c}. \qquad (27)
$$

While the linearization of the Hill function $k_s(q)$ is the one presented in Eq.9, we have to introduce the linearization of $k_r(q,j)$:

$$
\begin{aligned}
k_r(q,j) \quad \sim \quad & k_r(q,j)|_{<q>,<j>} + \partial_q k_r(q,j)|_{<q>,<j>}(q- <q>) \\
+ \quad & \partial_j k_r(q,j)|_{<q>,<j>}(j- <j>). \qquad (28)
\end{aligned}
$$

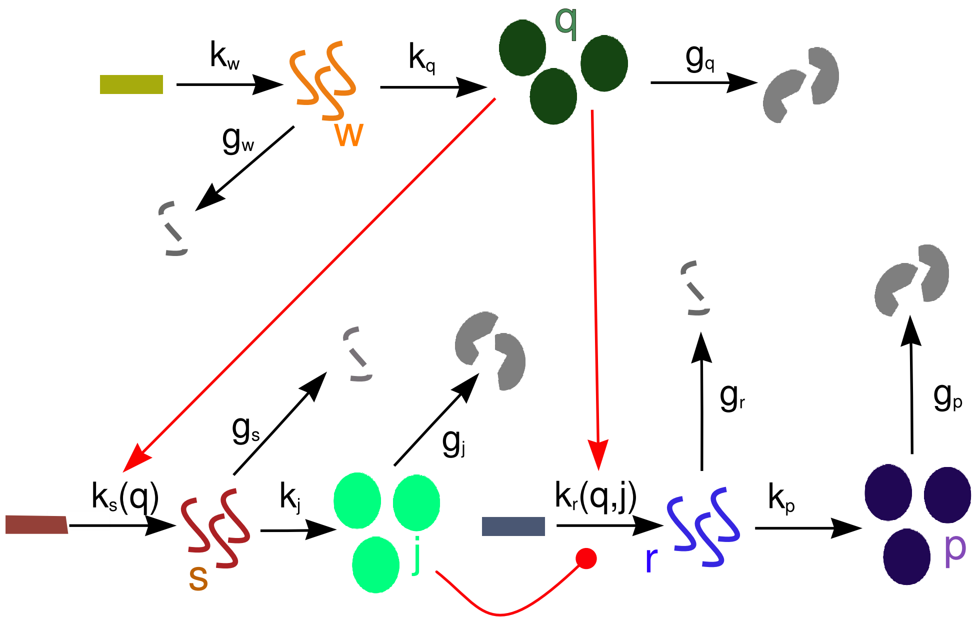


**Figure 13.** Scheme of a purely transcriptional incoherent FFL. Notations are the same of Fig.2. The only difference with respect to Fig.2 is the presence of a protein in the indirect pathway from the TF to target gene, therefore there are the additional reactions: translation of the protein $j$ from its mRNAs $s$ with rate $k_j$, and their degradation with rate $g_j$. The repressive action on the target is at the level of transcription in this case (represented by the red rounded end line), resulting in a rate of target transcription $k_r(q,j)$ which is a function of the number of proteins $j$ and master TFs $q$.

Therefore we can define:

$$\begin{aligned} k_r^0 &= k_r(q,j)|_{<q>,<j>} - \partial_j k_r(q,j)|_{<q>,<j>} < j > - \partial_q k_r(q)|_{<q>,<j>} < q > \\ k_r^1 &= \partial_q k_r(q,j)|_{<q>,<j>} \\ k_r^2 &= \partial_j k_r(q,j)|_{<q>,<j>}. \end{aligned} \quad (29)$$

Using the linearization just defined and the method of moment generating function described in section 2, the analytical expression of $< p >$ and $CV_p$ can be obtained.

## 5.2 Constraints on parameters for a comparison with miRNA-mediated FFLs

As stated in the main text, in order to make an unbiased comparison of the noise properties of these two circuits, the corresponding models must be constrained to produce the same amount of target proteins. There are several possible ways of putting this constraint, due to the fact that there are two additional parameters in the transcriptional FFL ($k_j$ and $g_j$) and therefore two supplementary degrees of freedom. In fact, a constraint can be inserted for example in the Hill function of target activation (tuning $h_j$) or in the rate of $s$ transcription $k_s(q)$, choosing accordingly the values of $k_j$ and $g_j$. This variety of options introduces some arbitrariness in the comparison. Our criterion is to keep the shared parameters to the same values (i.e. repression/activation efficiencies and production/degradation rates) and choose the two additional ones to make equal the average amount of repressor proteins $< j >$ in the transcriptional case to the average amount of miRNAs $< s >$ in the mixed circuit. With this choice we end up with the

same average amount of repressors in the two circuit versions ($< j >=< s >$), with the same efficiency of repression ($h_j = h_p$), and therefore with same impact on the target expression (making equal the amount of target proteins produced by the two circuits). To implement this constraint, in the transcriptional FFL the rate of translation $k_j$ must simply equal the rate of degradation $g_j$ (that we assume equivalent to the other protein degradation rates $g_j = g_q = g_p$). As a result, the average number of proteins $j$ which is produced from a single mRNA is forced to $b = k_j/g_s = g_j/g_s$. $b$ represents the translational burst size and, as discussed in the main text, it is a critical quantity in determining the noise level. As reported in [1], the fluctuations in the concentration of a single gene product can be expressed as:

$$CV^2 = \frac{1}{< p >}\left(\frac{b}{1+\eta} + 1\right). \tag{30}$$

Therefore, the noise level is dependent on the translational burst size (where $\eta$ is the ratio of protein to mRNA lifetime).
We report the parameter values used in the analysis summarized in Figure 8 of the main text: $k_j = g_j = g_q = g_p = 0.002; g_w = g_s = g_r = 0.006, k_r = 0.8; k_s = 0.5; c = 2; h_r = 200; h_s = 200; k_p = 0.04$. For these values, the translational burst size $b$, compatible with constraints, is $b = 0.33$, which is considerably smaller than expected in eukaryotes. In conclusion, to satisfy the constraints we are probably underestimating the noise introduced by the supplementary translation step required in a purely transcriptional FFL. This is why we expect that a miRNA-mediated FFL can overcome in noise-buffering efficiency its purely transcriptional counterpart even more than reported in Figure 8 of the main text.

# 6 Cross-talk between miRNA targets

## 6.1 Stoichiometric vs catalytic model of miRNA action

We start from a mass-action model for miRNA-mediated FFLs where we introduce explicitily a paramenter $\alpha$ representing the degree of catalyticity of miRNA action on targets. This type of description was introduced by Levine et al [9] and it will be straightforwardly applied to the FFL case in the following:

$$\begin{aligned}
\frac{dw}{dt} &= k_w - g_w w \\
\frac{dq}{dt} &= k_q w - g_q q \\
\frac{ds}{dt} &= k_s(q) - g_s s - (k_+ rs - k_- c) + (1-\alpha)\beta c \\
\frac{dr}{dt} &= k_r(q) - g_r r - (k_+ rs - k_- c) \\
\frac{dc}{dt} &= (k_+ rs - k_- c) - \beta c \\
\frac{dp}{dt} &= k_p r - g_p p,
\end{aligned} \tag{31}$$

where $c$ is the concentration of miRNA-mRNA complexes, $k_+$ is the probability of miRNA-mRNA association, $k_-$ the probability of dissociation of the complex $c$, which can degrade with rate $\beta$. The parameter $\alpha$ represents the probability that degradation of the mRNA in the complex is accompained by degradation of the miRNA. As discussed in [9], it is a measure of how much the miRNA action is catalytic. In this section, the variables that describe the state of the system ( $\{w, q, r, s, c, p\}$) are continuous variables, representing the average number of the various molecular species (we are omitting

the notation $< .. >$ for averages). Since we are interested in steady state properties, we can simplify the model equilibrating the $c$ complex dynamics:

$$\begin{aligned}
\frac{dw}{dt} &= k_w - g_w w \\
\frac{dq}{dt} &= k_q w - g_q q \\
\frac{ds}{dt} &= k_s(q) - g_s s - \alpha\gamma rs \\
\frac{dr}{dt} &= k_r(q) - g_r r - \gamma rs \\
\frac{dp}{dt} &= k_p r - g_p p, \qquad (32)
\end{aligned}$$

where $\gamma = \beta k_+/(k_- + \beta)$. The limit of $\alpha = 0$ implies that for each degradation event of $c$ complexes, none of the miRNAs is lost. This corresponds to a simplification of the model presented in section 3 of Text S1: the rate of mRNA degradation is supposed to be a linear function of miRNA concentration, instead of a nonlinear Hill function. The opposite situation of $p = 1$ reproduces the stoichiometric model presented in Eq.25 (apart from the sostitution $\gamma \to k_{rs}$).
It is straightforward to generalize this description to the case of two miRNA targets, adding an equation describing the dynamics of a second target which is independently transcribed:

$$\begin{aligned}
\frac{dw}{dt} &= k_w - g_w w \\
\frac{dq}{dt} &= k_q w - g_q q \\
\frac{ds}{dt} &= k_s(q) - g_s s - \alpha(\gamma_1 rs + \gamma_2 r_2 s) \\
\frac{dr}{dt} &= k_r(q) - g_r r - \gamma_1 rs \\
\frac{dr_2}{dt} &= k_{r_2} - g_{r_2} r_2 - \gamma_2 r_2 s \\
\frac{dp}{dt} &= k_p r - g_p p. \qquad (33)
\end{aligned}$$

The analytical solutions can be found easily at the steady state. This is the complete effective model presented partially in the main text. In the following the coupling constants between miRNAs and the mRNAs transcribed from the two target genes will be assumed equal ($\gamma_1 = \gamma_2 = \gamma$).

## 6.2 Details on the model setting

In this section we present a detailed view of the model setting and parameter values used for the analysis regarding the target cross-talk presented in the main text. We focus specifically on the minimal assumptions and on the parameter values used to achieve the results in Figure 9.

### 6.2.1 Setting for Figure 9 B

The solution for $< p >$ of Eqs. 33 at the steady state depends on $\alpha$. Therefore, in order to evaluate the impact of the dilution effect for different mechanisms (stoichiometric/catalytic) of miRNA repression, we choose for each $\alpha$ the corresponding $\gamma$ value that leads to the same mean amount of target proteins $< p >$.

Qualitatively, in a catalytic model ($\alpha = 0$) the miRNAs are more efficient since they can affect several target mRNAs without being degraded. Consequently, as $\alpha$ decreases the $\gamma$ value must be decreased so as to mantain the same target level expression. This is the constraint that makes unique the starting point (for $k_{r_2} = 0$) of the curves corresponding to different $\alpha$ values in Fig. 9 of the main text. Concerning the other parameter values, in Fig. 9 they are fixed to: $k_q = 0.19s^{-1}; g_q = g_p = 0.002s^{-1}; g_w = g_r = g_s = g_{r_2} = 0.006s^{-1}; k_w = 0.0126s^{-1}; k_r = k_s = 0.8s^{-1}; k_p = 0.04s^{-1}; c = 2; h_r = h_s = 200$, while the value of $\gamma = 0.00011s^{-1}$ is assigned to the catalytic model ($\alpha = 0$) - and it corresponds approximately to the optimal buffering value-, while the $\gamma$ values for the other $\alpha$ models can be calculated as described above.

#### 6.2.2 Setting for Figure 9 C

As a first approximation we assume, for the sake of simplicity, that the expression of the second target is not regulated by any TF. Therefore, it is a simple birth-death process, with transcription rate $k_{r_2}$ and degradation rate $g_{r_2}$ (assumed unique for both targets $g_{r_2} = g_r$). Since the target embedded in the FFL is regulated by the TFs $q$, $k_r(< q >)$ can be used as the effective rate of transcription, to be compared with $k_{r_2}$ of the second target. Indeed, $k_r(< q >)$ represents the average rate at which the joint target is transcribed.

#### 6.2.3 Setting for Figure 9 D

In this subsection we shall introduce a simple strategy to tune the second target fluctuations and analyze their impact on noise buffering efficiency. The proposed strategy is in perfect analogy with the one explained in section "The incoherent feedforward loop is effective in reducing extrinsic fluctuations" of the main text. In brief, we add an independent TF $q'$ which activates the transcrition of the second target. Its rates of transcription $k'_w$ and translation $k'_q$ are chosen so as to produce the same mean amount of protein of the other activator ($< q >=< q' >$). Therefore, the effective mean rates of transcription of both miRNA targets turn out to be equal. Changing the ratio $k'_w/k'_q$ while keeping costant the product $k'_w k'_q$ allows us to vary the second target fluctuations without altering its mean level.

## 7 Noise reduction and signaling sensitivity

Biological systems present the apparently contraddictory need for both high sensitivity to external signals both homeostatic controls, depending on the specific function in analysis. Indeed, while one essential feature of signal transduction systems is the amplification of small changes in input signals [12], the reliable celullar functioning in a fluctuating environment lays on multiple homeostatic controls (the most evident is temperature control in mammals). Similarly, at the level of genetic networks there is an interplay between sensitivity to changes in the input signal and the ability to buffer stochastic fluctuations. An increase in sensitivity to a signal results in an elevated exposure to its fluctuations, as shown for linear cascades of regulations [12,13]. More recently the sensitivity/noise-buffering analysis has been extended to small genetic circuits, including feedback and feedforward loops [4]. The working hypothesis of the authors is that the main function of a genetic circuit is to maximize the amplification of input signals. We argue that while this can be often the case, some circuits can have evolved to mantain reliably a functional steady state, even at the expense of a loss of sensitivity (and even thanks to that loss), to implement in other words a homeostatic control.

Following [4], the steady state sensitivity can be defined as the relative response in output that follows a change in the input. In the contest of incoherent FFLs (scheme in Fig. 2) we can consider as input the mean number of TFs $< q >$ and as output the consequent level of target proteins $< p >$. Following these definitions, the susceptibility takes the form:

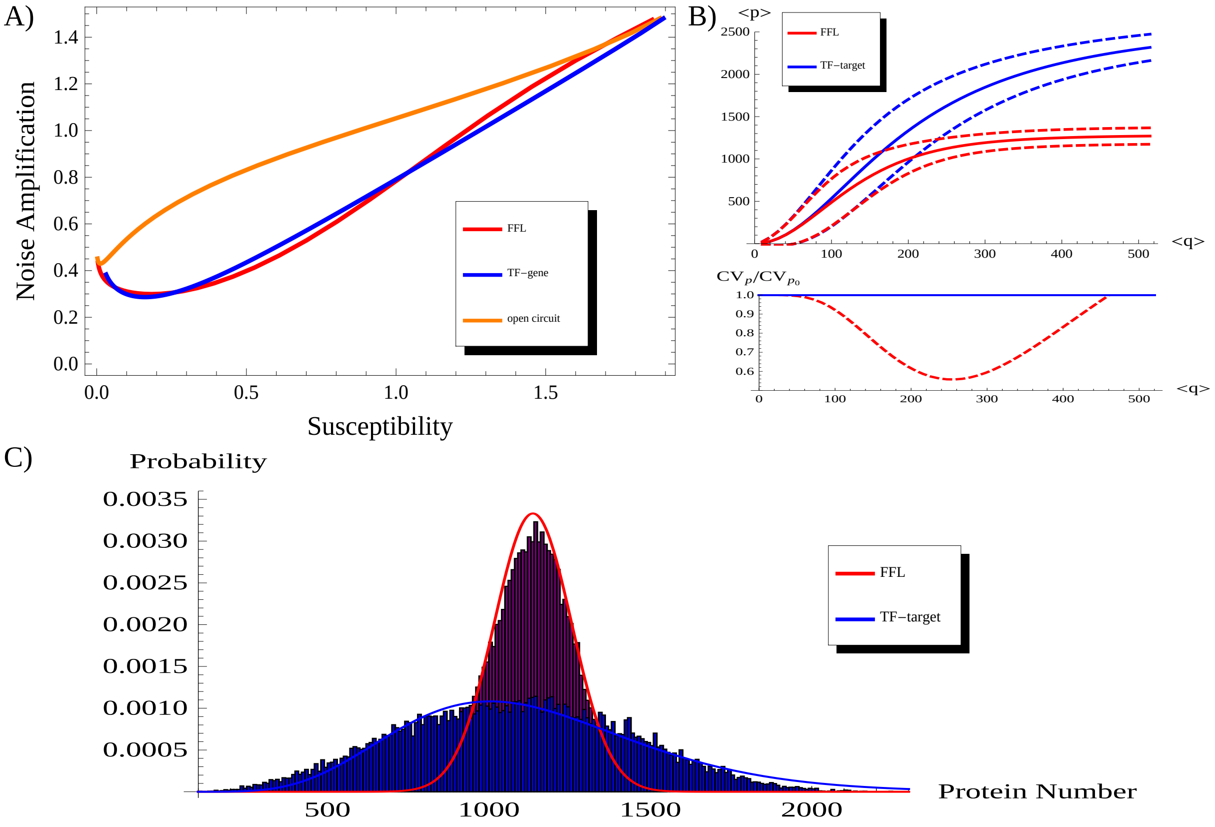


**Figure 14.** (A) Noise amplification versus susceptibility for the three circuits: a miRNA-mediated incoherent FFL, a TF-target regulation and an open circuit. The parameter values that are fixed are those reported in caption of Fig.3 of the main text (unless $k_q = 0.19s^{-1}$ higher than in Fig.3 to increase the TF fluctuations). (B) The upper panel shows the fold change in the target level in response to a fold change in the TF level for the miRNA-mediated incoherent FFL and the TF-target linear circuit. Continuous lines represent the behaviour of mean values while dashed lines are depicted at a distance from the mean equal to one standard deviation. In the lower panel the noise reduction $CV_p/CV_{p_0}$ is depicted in same range of $< q >$.(C) The probability distribution of protein number for the two circuits (miRNA-mediated FFL and TF-gene). In this case the two regulative circuits are constrained to produce an equal mean amount of target proteins. The same steady state is achieved with a strikingly different control of fluctuations by the two circuitries. Histograms are the result of Gillespie simulations while continuous lines are empirical distributions (gaussian for the FFL and gamma for the TF-gene) with mean and variance predicted by the analytical model.

$$susceptibility = \frac{< q >}{< p >} \frac{d \ \ < p >}{d \ \ < q >} = \frac{d \ ln(< p >)}{d \ ln(< q >)}. \tag{34}$$

As a measure of the quantity of noise propagating through the circuit, the noise amplification measure $\eta$ can be introduced [4]:

$$\eta = \frac{CV_p}{CV_q}, \tag{35}$$

defined as ratio between output and input noise. As shown in Fig. 14 A, the incoherent miRNA-mediated FFL presents an interplay between noise amplification and susceptibility very similar to that of a gene only activated by a TF, while the same fine-tuning implemented using an independent miRNA would imply a more severe interplay. Therefore, the noise buffering function demonstrated in this paper is achieved at the expense of steady-state sensitivity: given a fixed value of susceptibility, the FFL and the TF-gene linear circuit lead to a similar degree of noise amplification, while when the noise is buffered by the FFL there must be a loss of target susceptibility. Indeed, the fold change in target expression, that follows a change in the TF mean level $< q >$, is reduced precisely in the region where the noise control is implemented (see Fig.14 B). However, we propose that this is precisely the behaviour needed for a homeostatic control. The output is highly sensitive to changes in the input concentration until a finely tuned steady state is reached, then this functional steady state is kept robust to input fluctuations even if at the expense of a sensitivity loss. The same steady state could be reached more simply without any miRNA regulation, tuning the TF concentration in a TF-gene circuit, so as to conserve a high sensitivity. However, in this case the equilibrium level would be affected by strong fluctuations propagating from the upstream factor, as clearly shown in Fig. 14 B.
In conclusion, if the sensitivity is the function that have to be maximized, as it is probably the case in signaling systems, incoherent FFLs (and miRNA mediated ones) are outperformed by other circuits (like those making use of positive feedbacks loops [4]) that support less noise amplification at a fixed susceptibility. However, in different biological contests a high sensitivity could be important only until a functional steady state is reached. Then a homeostatic control can be required for keeping the reached level constant in presence of noisy upstream regulators and miRNA-mediated FFLs seems properly designed for this aim. The proposed functioning is also in agreement with the idea of fine-tuning: when the target expression is switched on by a rise in TF concentration, the maintenance of its level into a narrow functional range can be more important than a reliable transmission of further incoming small signals. A role of miRNA regulation in homeostasis is in line with the observation that miRNAs are often involved in signaling networks to ensure homeostatic controls (see for example [14]).

# 8 Effects of possible delays in miRNA production.

The common lore is that a RNA based post-transcriptional regulation can have a faster action on a target gene expression with respect to TF regulation [20, 21]. Indeed, a TF must be transported back to the nucleus and find its target promoter to exert its regulative role. However there is a lack of data to support quantitatively this assumption and the biogenesis of miRNA actually requires several processing steps. The time needed for the miRNA to be processed, loaded in RISC and in general to become active can introduce a delay between its transcription and its effect on targets. Therefore it could be interesting to consider possible effects of this time delay on the noise buffering function of mixed FFLs. While in the model presented in the main text the miRNA is supposed to act on its target instantaneously, in this section we present results of simulations performed taking into account the time-delay that can arise from miRNA processing. More specifically the time-delay has been inserted in the Hill function of regulation of the target translation, mimicking the time required for miRNA activation. With this simulation procedure, for each chosen set of parameter values it is possible to establish the threshold in delay time below which the circuit is able to reduce target fluctuations.
In Figure 15 the noise reduction achieved with a FFL ($CV_p/CV_{p0}$) is reported as a function of the time required for miRNA activation. The time-delay is expressed in unit of protein half-life, chosen as a reference since it represents the longest time-scale in the system. The ability of the circuit in filtering out fluctuations relies on the correlation between miRNA and target mRNA fluctuations, therefore an eventual time-delay in miRNA action can negatively affect the noise buffering. More specifically, with the parameter values of Fig.3-4 of the main text, the incoherent FFL is no more able to reduce target fluctuations if the delay is longer than approximately 3 protein half-life (see Fig 15). As the processing

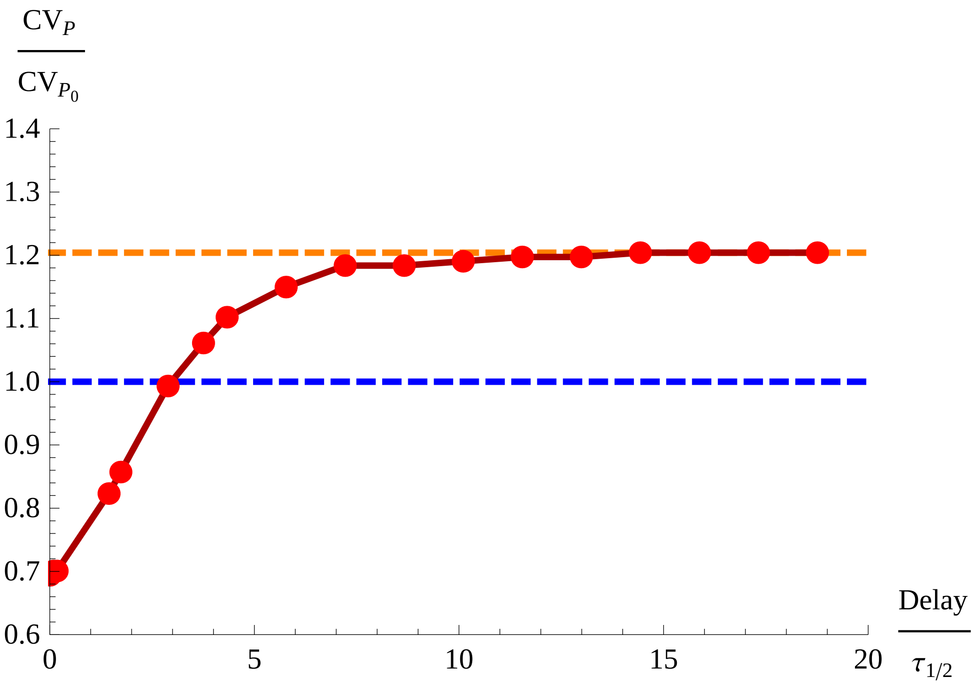


**Figure 15.** The effect on noise-buffering efficiency of a time-delay between miRNA transcription and miRNA repressive action. The values of parameters are the ones in caption of Figure 3 in the main text. The target noise reduction $CV_p/CV_{p_0}$ is measured as a function of the length of the time-delay expressed in unit of protein half-life $\tau_{1/2}$. $CV_{p_0}$ is the constitutive noise of a TF-gene circuit without the miRNA regulation. Until the delay length approaches approximately 3 protein half-lives, the FFL is still able to filter out fluctuations. After that the noise level tends to the value achieved with an open circuit (dashed orange line) in which miRNAs and target mRNAs have uncorrelated fluctuations in their level. Dots are the result of Gillespie's simulations with the full nonlinear dynamics.

time becomes longer and longer, miRNA fluctuations lose any correlation with the target ones and the target noise approches the value corresponding to the open circuit case (dashed orange line in Fig. 15). In conclusion we showed that a significant time-delay between miRNA transcription and target repression can compromise the noise-buffering function. When quantitative measures of the time required for transport and processing of miRNAs and proteins will be available, it will be possible to precisely evaluate the degree of reduction of target fluctuations inserting the appropriate delays in the Hill function of regulations of our theoretical model (even if at the expense of its analytical tractability).

# 9 Bioinformatical analysis of miRNAs involved in FFLs in the human mixed network.

Although miRNA mediated FFLs have been shown to be overrepresented in real mixed networks with respect to randomized networks [15–18], it is equally important to establish the numerical fraction of miRNAs and miRNA targets that are actually involved in these circuits, to better highlight the effective biological relevance of miRNA-mediated FFLs. To this aim we take advantage of the genome-wide survey of human miRNA-mediated FFLs previously developed by our group [18], based on a search for

overrepresented motif in human and mouse promoters and 3'-UTRs. Of the 464 miRNAs annotated as KNOWN-KNOWN in the Ensembl database (release 46) [19], using the filters and the software setup of [18] 193 were selected to form the post-transcriptional network (miRNA-target interactions). Integrating this network with the transcriptional one (with TF-target and TF-miRNA interactions), 133 miRNA have been significantly associated to at least one (usually more than one) of the 5030 mixed FFLs found in the human regulatory network (see [18] for more details). Therefore miRNAs, at least in the database considered, seem often involved in FFL circuits. Since each miRNA can regulate hundreds of targets it is also interesting to evaluate what fraction of its targets are part of FFLs. The results of this analysis are reported in the following Table where the total number of targets and the number of targets in a FFL is presented for each miRNA embedded in a FFL . While some miRNAs preferentially regulate genes through a FFL topology this is clearly not a general trend, further confirming the importance of considering the possible cross-talk between miRNA targets (as discussed in the main text).
However it is important to notice that the proposed results suffer some limitations. Firstly we cannot distinguish incoherent from coherent FFLs since sequence analysis allows the identification of putative interactions but cannot establish if they are positive or negative. Secondly the proposed regulations should be considered as potential interactions because they represent purely bioinformatic predictions and furthermore the miRNA and its targets could be expressed preferentially in different tissues or at different times. In this case the eventual cross-talk would be limited among co-expressed targets. In spite of the reported limitations, the data presented here point out that miRNA-mediated FFLs can actually represent an often exploited regulative circuitry, further suggesting their importance in real networks of gene regulations.

| miRNA gene | Num. of targets | Num. of targets in FFLs | Percentage |
|---|---|---|---|
| hsa-miR-129 | 44 | 36 | 81.8 % |
| hsa-miR-148b | 127 | 84 | 66.1 % |
| hsa-miR-149 | 55 | 36 | 65.5 % |
| hsa-miR-449b | 55 | 34 | 61.8 % |
| hsa-let-7a | 83 | 51 | 61.4 % |
| hsa-miR-199a* | 138 | 84 | 60.9 % |
| hsa-miR-125b | 150 | 90 | 60.0 % |
| hsa-miR-199a | 41 | 24 | 58.5 % |
| hsa-miR-101 | 105 | 61 | 58.1 % |
| hsa-miR-205 | 38 | 22 | 57.9 % |
| hsa-miR-31 | 35 | 20 | 57.1 % |
| hsa-miR-203 | 51 | 29 | 56.9 % |
| hsa-miR-30c | 155 | 87 | 56.1 % |
| hsa-miR-425-3p | 50 | 28 | 56.0 % |
| hsa-miR-9 | 106 | 59 | 55.7 % |
| hsa-miR-296 | 69 | 38 | 55.1 % |
| hsa-miR-194 | 90 | 49 | 54.4 % |
| hsa-miR-181d | 120 | 64 | 53.3 % |
| hsa-miR-219 | 123 | 65 | 52.8 % |
| hsa-miR-32 | 148 | 78 | 52.7 % |
| hsa-miR-9* | 100 | 52 | 52.0 % |
| hsa-miR-148a | 91 | 47 | 51.6 % |
| hsa-miR-24 | 107 | 54 | 50.5 % |
| hsa-miR-133b | 40 | 20 | 50.0 % |
| hsa-miR-499 | 40 | 20 | 50.0 % |
| hsa-miR-30a-3p | 48 | 23 | 47.9 % |
| hsa-miR-218 | 83 | 39 | 47.0 % |
| hsa-miR-375 | 113 | 53 | 46.9 % |
| hsa-miR-223 | 145 | 67 | 46.2 % |
| hsa-miR-100 | 46 | 21 | 45.7 % |
| hsa-miR-214 | 62 | 28 | 45.2 % |
| hsa-miR-10a | 39 | 17 | 43.6 % |
| hsa-miR-1 | 46 | 20 | 43.5 % |
| hsa-miR-130a | 127 | 55 | 43.3 % |
| hsa-miR-30a-5p | 155 | 67 | 43.2 % |
| hsa-miR-802 | 76 | 31 | 40.8 % |
| hsa-miR-26a | 129 | 52 | 40.3 % |
| hsa-miR-23a | 152 | 60 | 39.5 % |
| hsa-miR-99a | 46 | 18 | 39.1 % |
| hsa-miR-126* | 181 | 70 | 38.7 % |
| hsa-miR-330 | 50 | 19 | 38.0 % |
| hsa-miR-135b | 103 | 39 | 37.9 % |

| miRNA gene | Num. of targets | Num. of targets in FFLs | Percentage |
|---|---|---|---|
| hsa-miR-133a | 40 | 15 | 37.5 % |
| hsa-miR-155 | 100 | 37 | 37.0 % |
| hsa-miR-126 | 109 | 40 | 36.7 % |
| hsa-miR-140 | 106 | 38 | 35.8 % |
| hsa-miR-506 | 127 | 45 | 35.4 % |
| hsa-miR-99b | 46 | 16 | 34.8 % |
| hsa-miR-202 | 88 | 30 | 34.1 % |
| hsa-miR-135a | 103 | 35 | 34.0 % |
| hsa-let-7f | 83 | 28 | 33.7 % |
| hsa-miR-16 | 57 | 19 | 33.3 % |
| hsa-let-7d | 90 | 29 | 32.2 % |
| hsa-let-7e | 127 | 40 | 31.5 % |
| hsa-miR-542-3p | 39 | 12 | 30.8 % |
| hsa-miR-206 | 46 | 14 | 30.4 % |
| hsa-miR-34b | 55 | 16 | 29.1 % |
| hsa-miR-34c | 55 | 16 | 29.1 % |
| hsa-miR-342 | 49 | 14 | 28.6 % |
| hsa-miR-363 | 84 | 24 | 28.6 % |
| hsa-miR-365 | 46 | 13 | 28.3 % |
| hsa-miR-27a | 104 | 29 | 27.9 % |
| hsa-miR-29a | 115 | 32 | 27.8 % |
| hsa-miR-19a | 145 | 39 | 26.9 % |
| hsa-miR-152 | 127 | 34 | 26.8 % |
| hsa-miR-199b | 41 | 11 | 26.8 % |
| hsa-miR-141 | 146 | 38 | 26.0 % |
| hsa-miR-212 | 58 | 15 | 25.9 % |
| hsa-miR-302c* | 93 | 24 | 25.8 % |
| hsa-miR-106a | 126 | 32 | 25.4 % |
| hsa-miR-17-5p | 126 | 32 | 25.4 % |
| hsa-miR-30e-5p | 155 | 39 | 25.2 % |
| hsa-miR-495 | 123 | 31 | 25.2 % |
| hsa-miR-144 | 146 | 36 | 24.7 % |
| hsa-miR-7 | 89 | 22 | 24.7 % |
| hsa-miR-20b | 126 | 31 | 24.6 % |
| hsa-miR-20a | 132 | 32 | 24.2 % |
| hsa-miR-103 | 97 | 23 | 23.7 % |
| hsa-miR-106b | 132 | 31 | 23.5 % |
| hsa-miR-367 | 111 | 26 | 23.4 % |
| hsa-miR-34a | 43 | 10 | 23.3 % |
| hsa-miR-193a | 112 | 26 | 23.2 % |
| hsa-miR-200c | 143 | 33 | 23.1 % |
| hsa-miR-189 | 35 | 8 | 22.9 % |

| miRNA gene | Num. of targets | Num. of targets in FFLs | Percentage |
|---|---|---|---|
| hsa-miR-93 | 83 | 19 | 22.9 % |
| hsa-miR-202* | 49 | 11 | 22.4 % |
| hsa-miR-451 | 45 | 10 | 22.2 % |
| hsa-miR-221 | 50 | 11 | 22.0 % |
| hsa-miR-222 | 50 | 11 | 22.0 % |
| hsa-miR-138 | 60 | 13 | 21.7 % |
| hsa-miR-302b | 134 | 29 | 21.6 % |
| hsa-miR-302c | 134 | 29 | 21.6 % |
| hsa-miR-302d | 134 | 29 | 21.6 % |
| hsa-miR-299-5p | 108 | 23 | 21.3 % |
| hsa-miR-182 | 80 | 17 | 21.2 % |
| hsa-miR-142-5p | 57 | 12 | 21.1 % |
| hsa-miR-369-3p | 101 | 21 | 20.8 % |
| hsa-let-7b | 83 | 17 | 20.5 % |
| hsa-miR-494 | 122 | 24 | 19.7 % |
| hsa-miR-183 | 92 | 18 | 19.6 % |
| hsa-miR-505 | 51 | 10 | 19.6 % |
| hsa-miR-377 | 82 | 16 | 19.5 % |
| hsa-miR-96 | 133 | 26 | 19.5 % |
| hsa-miR-195 | 57 | 11 | 19.3 % |
| hsa-miR-497 | 57 | 11 | 19.3 % |
| hsa-miR-30e-3p | 48 | 9 | 18.8 % |
| hsa-miR-381 | 165 | 31 | 18.8 % |
| hsa-miR-142-3p | 127 | 23 | 18.1 % |
| hsa-miR-139 | 34 | 6 | 17.6 % |
| hsa-miR-30b | 155 | 27 | 17.4 % |
| hsa-miR-30d | 155 | 27 | 17.4 % |
| hsa-miR-302b* | 76 | 13 | 17.1 % |
| hsa-miR-487b | 83 | 14 | 16.9 % |
| hsa-miR-369-5p | 90 | 15 | 16.7 % |
| hsa-miR-409-5p | 80 | 13 | 16.2 % |
| hsa-miR-410 | 133 | 21 | 15.8 % |
| hsa-miR-329 | 93 | 14 | 15.1 % |
| hsa-miR-151 | 70 | 10 | 14.3 % |
| hsa-miR-412 | 42 | 6 | 14.3 % |
| hsa-miR-25 | 74 | 10 | 13.5 % |
| hsa-miR-192 | 45 | 6 | 13.3 % |
| hsa-miR-496 | 113 | 14 | 12.4 % |
| hsa-miR-153 | 100 | 9 | 9.0 % |
| hsa-miR-15a | 57 | 5 | 8.8 % |
| hsa-miR-217 | 102 | 9 | 8.8 % |
| hsa-miR-323 | 57 | 5 | 8.8 % |

| miRNA gene | Num. of targets | Num. of targets in FFLs | Percentage |
|---|---|---|---|
| hsa-miR-484 | 100 | 6 | 6.0 % |
| hsa-miR-26b | 129 | 7 | 5.4 % |
| hsa-miR-146b | 40 | 2 | 5.0 % |
| hsa-miR-200a* | 90 | 3 | 3.3 % |
| hsa-miR-200a | 146 | 3 | 2.1 % |
| hsa-miR-200b | 143 | 2 | 1.4 % |
| hsa-miR-429 | 108 | 1 | 0.9 % |

# References


1. Thattai M and van Oudenaarden A (2001) Intrinsic noise in gene regulatory networks. Proc Natl Acad Sci USA 98: 8614-8619.

2. Komorowski M, Miekisz J and Kierzek AM (2009) Translational repression contributes grater noise to gene expression than transcriptional repression. Biophys J 96(2): 372-84.

3. Mangan S and Alon U (2003) Structure and function of the feed-forward loop network motif. Proc Natl Acad Sci USA 100: 11980-11985.

4. Hornung G and Barkai N (2008) Noise propagation and signaling sensitivity in biological networks: a role for positive feedback. PLoS Comput Biol 4(1): e8.

5. Valencia-Sanchez MA, Liu J, Hannon GJ, Parker R (2006) Control of translation and mRNA degradation by miRNAs and siRNAs. Genes Dev 20: 515-524.

6. Pillai RS, Bhattacharyya SN and Filipowicz W (2007) Repression of protein synthesis by miRNAs: how many mechanisms? Trends Cell Biol 17: 118-126.

7. Filipowicz W, Bhattacharyya SN, Sonenberg N (2008) Mechanisms of post-transcriptional regulation by microRNAs: are the answer in sight? Nat Rev Genet 9: 102-114.

8. Metha P, Goyal S and Wingreen NS (2008) A quantitative comparison of sRNA-based and protein-based gene regulation. Mol Syst Biol 4: 221.

9. Levine E, Kuhlman T, Zhang Z, Hwa T (2007) Quantitative characteristics of gene regulation by small RNA. PLoS Biology 9: 229.

10. Shimoni Y, Friedlander G, Hetzroni G, Niv G, Altuvia S, Biham O, Margalit H (2007) Regulation of gene expression by small non-coding RNAs: a quantitative view. Mol Syst Biol 3: 138.

11. Elf J, Paulsson J, Berg OG, Ehrenberg (2005) Mesoscopic kinetics and its applications in protein synthesis. In *Topics in Current Genetics: Systems Biology: Definitions and Perspectives*, Alberghina L, Westerhoff HV (eds), pp 95-116. Berlin, Germany: Springer-Verlag.

12. Shibata T and Fulimoto K (2005) Noisy signal amplification in ultrasensitive signal transduction. Proc Natl Acad Sci USA 102: 331-336.

13. Hooshangi S, Thiberge S, Weiss R (2005) Ultrasensitivity and noise propagation in a synthetic transcriptional cascade. Proc Natl Acad Sci U S A 102: 3581-3586.

14. Inui M, Martello G e Piccolo S. (2010) MicroRNA control of signal transduction. Nat Rev Mol Cell Biol 11: 252-63.

15. Shalgi R, Lieber D, Oren M and Pilpel Y (2007) Global and local architecture of the mammalian microRNA-transcription factor regulatory network. PLoS Comput Biol 3(7): e131.

16. Tsang J, Zhu J and van Oudenaarden A (2007) MicroRNA-mediated feedback and feedforward loops are recurrent network motifs in mammals. Mol Cell 26: 753-767.

17. Yu X, lin J, Zack DJ, Mendell JT and Qian J (2008) Analysis of regulatory network topology reveals functionally distinct classes of microRNAs. Nucleic Acids Res 36: 6494-6503.

18. Re A, Corá D, Taverna D and Caselle M (2009) Genome-wide survey of microRNA-transcription factor feed-forward regulatory circuits in human. Mol BioSyst 5: 854-867.

19. Hubbard T, Aken B, Beal K, Ballester B and Caccamo M et al. (2007) Ensembl 2007. Nucleic Acids Res 35: D610-D617.

20. Li X, Cassidy JJ, Reinke CA, Fischboeck S and Carthew RW (2009) A microRNA imparts robustness against environmental fluctuation during development. Cell 137: 273-282.

21. Mukherji S and van Oudenaarden A (2009) Synthetic biology: understanding biological design from synthetic circuits. Nature Reviews 10: 859-871.
